# Panaxadiol and Panaxatriol Derivatives as Anti-Hepatitis B Virus Inhibitors

**DOI:** 10.1007/s13659-014-0018-2

**Published:** 2014-05-13

**Authors:** Hao Chen, Li-Jun Wang, Yun-Bao Ma, Xiao-Yan Huang, Chang-An Geng, Xue-Mei Zhang, Ji-Jun Chen

**Affiliations:** 1 State Key Laboratory of Phytochemistry and Plant Resources in West China, Kunming Institute of Botany, Chinese Academy of Sciences, Kunming, 650201 People’s Republic of China; 2 University of Chinese Academy of Sciences, Beijing, 100049 People’s Republic of China

**Keywords:** Chemical modification, Panaxadiol and panaxatriol derivatives, Anti-HBV activity, Structure–activity relationships

## Abstract

**Abstract:**

28 Derivatives of panaxadiol (PD) and panaxatriol were synthesized and evaluated for their anti-HBV activity on HepG 2.2.15 cells, of which 17 derivatives inhibited HBV DNA replication. Compounds **4**, **9**, **10**, **14**, and **15** showed moderate activity against HBV DNA replication with IC_50_ values ranged from 7.27 to 28.21 μM compared with PD. In particular, 3-*O*-2′-thenoyl panaxadiol (**4**) inhibited not only HBV DNA replication (IC_50_ = 16.5 μM, SI > 115.7) but also HBsAg (IC_50_ = 30.8 μM, SI > 62.0) and HBeAg (IC_50_ = 18.2 μM, SI > 105.14) secretions. Their structure–activity relationships were discussed for guiding future research toward the discovery of new anti-HBV agents.

**Graphical Abstract:**

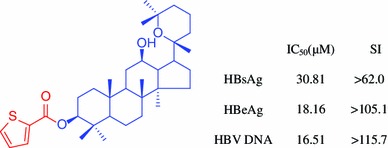

**Electronic supplementary material:**

The online version of this article (doi:10.1007/s13659-014-0018-2) contains supplementary material, which is available to authorized users.

## Introduction

Hepatitis B virus (HBV) infection is a serious health problem all over the world. There are about 350 million chronically infected individuals with the risk of approaching liver cirrhosis and hepatocellular carcinoma [1]. The current therapies for HBV infection involve immunomodulators, interferon-*α*, polyethylene glycol interferon-*α*, and nucleoside drugs, and are unsatisfactory due to high recurrence, drug resistance and inevitable side effects including influenza-like illness, myalgia, headache, reduction of neutrophilic granulocyte and blood platelet, etc. [2–5]. Therefore, it is interesting to explore novel anti-HBV agents with novel antiviral targets and mechanisms.

**Fig. 1:**
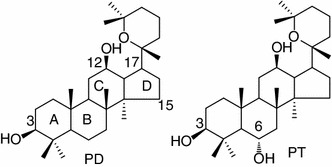
Panaxadiol (PD) and panaxatriol (PT)

**Scheme 1:**
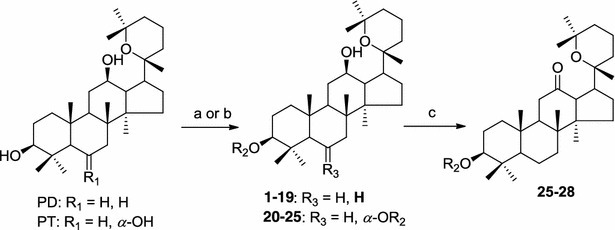
Synthesis of compounds **1**–**28**. Reagents and conditions: **a** corresponding acids, DMAP, DCC, CH_2_Cl_2_, rt; **b** anhydride, DMAP, anhydrous pyridine, reflux. **c** Jones reagent, acetone, rt

Natural products offer many opportunities to find lead compounds for drug discovery [6–10]. Dammarane triterpenes and their derivatives have antiviral and hepatoprotective potencies, as well as antitumor, hemolytic, antiplatelet, immunomodulatory, antioxidant and neuroprotective activities [11]. For example, chikusetsusaponin III reduced yield of herpes simplex virus type I with ID_50_ value of 29 μM [12]; panaxadiol (PD) derivatives incorporated with 2,2-dimethylsuccinyl group at C-3 and panaxatriol (PT) derivatives with same groups at C-3 and C-6, could inhibit HIV-1 protein proteases (IC_50_ = 2.7 ± 4.3 to 5.4 ± 3.8 μM) and HCV protein proteases (IC_50_ = 1.8 ± 2.6 to 30.4 ± 3.0 μM) [13]; furthermore, ginsenosides Rb_3_, Rc, Rd, XVII and notoginsenoside R_1_ from the flower buds of *Panax notoginseng* showed hepatoprotective activity against liver injury induced by D-galactosamine and lipopolysaccharide in mice [14]. Although derivatives of PD and PT (Fig. 1) exhibited antiviral and hepatoprotective effects, no report was concerned with their anti-HBV activity. As our ongoing study for searching anti-HBV inhibitors from natural resources, PD and PT were revealed to be active against HBV DNA replication with IC_50_ values of 148.15 and 668.60 μM but low SI values of 6.2 and 3.6 in our random assay. In order to increase the activity and safety, PD and PT were hybridized with heteroaromatic rings based on our previous experience from the modification on caudatin and hemslecin A [15, 16]. Consequently, 28 panaxadiol and panaxatriol analogues were synthesized by modifying on rings A, B and C. Herein, we described the synthesis, in vitro anti-HBV activity and structure–activity relationships (SARs) of these derivatives (Scheme 1).

## Results and Discussion

### Chemistry

The Steglich esterification condition was applied for synthesis of 3-*O*-substituted derivatives of PD and 3,6-*O*-disubstituted derivatives of PT in presence of 4-dimethylaminopyridine (DMAP), and *N*′,*N*′-dicyclohexylcarbodiimide (DCC). Derivatives (**2**–**13**, **20**–**21**) of PD and PT were also prepared with anhydrides under a catalytic amount of DMAP. There were no 12-*O*-substituted derivatives produced, of which the substituent position could be determined by the chemical shifts of derivatives at H-3 and H-12 in ^1^H NMR spectrum. For example, chemical shifts of H-3 and H-12 of PD appearred at *δ*_H_ 3.21 and 3.50 but at *δ*_H_ 4.42 and 3.52 of compound **1**. Furthermore, the hydroxyl group at C-12 of 3-*O*-substituted derivatives (**1**, **4** and **14**) were transformed as ketones by Jones reagent in order to disclose effects of hydroxyl groups.

### Anti-HBV Activity

PD, PT and their derivatives were evaluated for anti-HBV activities on HBsAg and HBeAg secretions, as well as HBV DNA replication on HepG 2.2.15 cells [9], and the results were summarized in Table 1. Accordingly, 4 active derivatives (**4**, **9**, **10** and **11**) inhibited HBsAg secretion with IC_50_ values ranged from 30.81 to 53.78 μM, and 3 active derivatives (**4**, **14** and **15**) suppressed HBeAg secretion with IC_50_ values ranged from 18.16 to 168.98 μM were obtained. Of the 17 active derivatives inhibiting HBV DNA replication, 9 derivatives showed IC_50_ values ranged from 7.27 to 86.28 μM. In particular, compound **4** possessed much better activity inhibiting not only HBV DNA replication (IC_50_ = 16.5 μM, SI > 115.7) but also HBsAg (IC_50_ = 30.8 μM, SI > 62.0) and HBeAg (IC_50_ = 18.2 μM, SI > 105.14) secretions, which is worth for further investigating.

**Table 1:** Anti-HBV activities and cytotoxicity of panaxadiol and panaxatriol derivatives in vitro

comp	*R*	CC_50_ (μM)	HBsAg		HBeAg		HBV DNA	
			IC_50_ (μM)	SI^a^	IC_50_ (μM)	SI	IC_50_ (μM)	SI
PD		923.15	>2282.61	NA	>2282.61	NA	148.15	6.2
PT		>2416.0	>2415.97	NA	>2415.97	NA	668.60	3.6
TA		8031.50	8031.50	NA	8031.50	NA	1771.65	NA
**1**		>2060.00	NA	NA	NA	NA	NA	NA
**2**		>2140.29	1253.09	1.7	>2140.29	NA	515.00	4.2
**3**		1184.87	833.89	1.4	1184.87	NA	50.27	23.6
**4**		>1910.21	30.81	>62.0	18.16	>105.1	16.51	>115.7
**5**		>2020.55	>2020.55	NA	>2020.55	NA	>505.14	NA
**6**		>1636.36	NA	NA	280.45	5.8	43.19	>37.9
**7**		>1689.79	NA	NA	NA	NA	NA	NA
**8**		>2003.55	>2003.55	NA	>2003.55	NA	500.89	4.0
**9**		44.36	43.26	1.0	NA	NA	9.02	4.9
**10**		25.93	45.59	NA	>1437.91	NA	7.27	3.6
**11**		1147.82	1147.82	NA	1147.82	NA	117.21	9.8
**12**		>1580.88	>1580.88	NA	>1580.88	NA	>1580.88	NA
**13**		1485.82	588.57	2.5	>2362.64	NA	>590.66	NA
**14**		179.00	198.75	NA	168.98	1.1	16.73	10.7
**15**		86.62	53.78	1.6	101.39	NA	28.21	3.1
**16**		>1701.39	>1701.39	NA	900.95	NA	51.93	>32.8
**17**		933.72	NA	NA	NA	NA	86.28	10.8
**18**		313.62	933.34	NA	NA	NA	124.29	2.5
**19**		>1728.97	>1728.97	NA	>1728.97	NA	>432.24	NA
**20**		>2104.3	>2104.3	NA	>2104.3	NA	545.37	NA
**21**		>1940.6	>1940.6	NA	>1940.6	NA	>485.1	>4.1
**22**		529.88	>1280.12	NA	>1280.12	NA	>320.03	NA
**23**		>2089.42	>2089.42	NA	>2089.42	NA	>2089.42	NA
**24**		1646.01	1646.01	NA	1068.93	1.54	411.50	1.66
**25**		>1500.00	>1500.00	NA	974.11	1.54	>375.00	4.0
**26**		>2280.0	NA	NA	NA	NA	NA	NA
**27**		>2155.8	NA	NA	NA	NA	NA	NA
**28**		>1940.6	>1940.6	NA	>1940.6	NA	>485.1	NA
Tf^b^		>1716.28	1389.42	>1.2	1237.86	>1.4	0.71	>2417.3

Values are means of two independent experiments

*HBsAg* hepatitis B surface antigen, *HBeAg* hepatitis B e antigen, *CC*_*50*_ 50 % cytotoxicity concentration in HepG 2.2.15 cells, *IC*_*50*_ 50 % inhibitory concentration, *NA* not available, *TA* thiophenezoic acid

^a ^SI (selectivity index) = CC_50_/IC_50_

^b^ Tenofovir as the positive control

Among the 3-*O*-substituted derivatives of PD, introduction of acetyl (**1**) and cyclopentanecarbonyl (**2**) into C-3 of PD reduced cytotoxicity and activities against HBV DNA replication. The 3-*O*-cyclopentanecarbonyl group of compound **2** was replaced by heteroatomic rings to generate 3-*O*-2′-furoyl (**3**), 3-*O*-2′-thenoyl (**4**) and 3-*O*-2′-nicotinoyl (**11**) derivatives providing better inhibitory activity with IC_50_ values of 50.27, 16.51 and 117.21 μM than PD and 3-*O*-benzoyl analogue (**8**). From the above analysis, it is suggested that heteroatomic rings played important roles in enhancing activity. Analogue **4** possessed the most active inhibition on HBsAg and HBeAg secretions, and HBV DNA replication with IC_50_ values of 30.81, 18.16 and 16.51 μM as well as SI values higher than 62.0, 105.1 and 115.7, indicating that 2-thenoyl group was preferable to suppress HBsAg and HBeAg secretions and HBV DNA replication, as well as improve safety. It is interesting that PD with moderate activity was esterified with inactive 2-thenoyl carboxylic acid (IC_50_ = 1771.65 μM) to produce an active hybrid **4**. In addition, 3-*O*-2′-(3′′-methyl) thenoyl (**5**), 3-*O*-2′-(3′′-chloro) thenoyl (**6**) and 3-*O*-(thianaphthene-2′-carbonyl) (**7**) derivatives exhibited less activity than 3-*O*-2′-thenoyl (**4**) analogue, indicating that substituents at 2-thenoyl moiety were unfavorable for anti-HBV activity.

3-*O*-Succinyl (**14**), 3-*O*-glutaryl (**15**) and 3-*O*-diglycolyl (**16**) analogues with free carboxyl groups were further prepared and showed better activity against HBV DNA replication than PD, of which compound **16** appeared the IC_50_ value of 51.93 μM and the SI value higher than 32.8, inferring that oxygen atom at side chain reduced cytotoxicity. Phenolic hydroxyl groups were introduced into the benzene ring of inactive compound **8** to offer derivatives **9** and **10** with 60-folds growth of inhibition on HBsAg secretion and HBV DNA replication, together with the increased cytotoxicity, indicating phenolic hydroxyl groups enhanced both activity and cytotoxicity.

Further modification on ring C of derivatives **1**, **4** and **14** by transforming the hydroxyl group at C-12 into the ketone group provided three inactive products **26**–**28** with IC_50_ values higher than 485.1 μM, demonstrating that hydroxyl group at C-12 is crucial for antiviral activity. Compared with PD, PT with one hydroxyl group at C-6 reduced activity, inferring that hydroxyl group at C-6 was detrimental to anti-HBV activity. This analysis was further supported by six 3,6-*O*-disubstituted derivatives (**20**–**25**) exhibited slight activity against HBV with IC_50_ values higher than 320.03 μM in contrast with PD derivatives which had same substituents, such as 2-furoyl, 2-thenoyl and succinyl groups.

## Conclusion

According to the results mentioned above, SARs were summarized as follows: (1) 2-thenoyl group at C-3 are favorable to enhance anti-HBV activity; (2) the hydroxyl group at C-12 is necessary for inhibitory activity; (3) hydroxyl group at C-6 was detrimental to anti-HBV activity. This study indicated that panaxadiol derivatives had moderate anti-HBV activity, and were worth further investigating for non-nucleoside anti-HBV drug candidates.

## Experimental Section

### General Experimental Procedures

MS and HRMS data were collected on Shimadzu liquid chromatography-mass spectrometry (LCMS)-ion trap (IT)-time of flight (TOF) (Shimadzu, Kyoto, Japan); All nuclear magnetic resonance (NMR) spectra were recorded on Bruker AM 400 (^1^H/^13^C) spectrometers (Bruker, Bremerhaven, Germany) with tetramethylsilane (TMS) as the internal standard; Column chromatography (CC): silica gel (200–300 mesh; Qingdao Makall Group Co., Ltd; Qingdao, China). All reactions were monitored using thin-layer chromatography (TLC) on silica gel plates. Corresponding substituted acids were purchased from Alfa Aesar (Tianjin, China) or J&K Scientific Ltd. (Beijing, China). Organic solvents were analytical reagent grade and purchased from Tianjin Chemical Reagent Co., Ltd (Tianjin, China).

Panaxadiol (PD) and Panaxatriol (PT) were isolated from *Panax notoginseng*. The powder of root and rhizoma of *P. notoginseng* (10.0 kg) was treated with 2 mol/L H_2_SO_4_ (15 L) under reflux for 1.5 h to give a reaction mixture in water, which extracted with chloroform (15 L × 3). The chloroform mixture was washed with water (30 L × 3), and then concentrated to dryness under reduced pressure. The chloroform part (1 kg) was chromatographed on silica gel column (3 kg, 17.5 × 35 cm, eluted with methanol - chloroform, 0:100–10:90, *v*/*v*) to provide fractions 3 and 5, which were purified by silica chromatograph (1.5 kg, 17.5 × 15 cm) and eluted with acetone - petroleum ether (15:85 and 20:80, respectively) to obtain PD (15 g) and PT (10 g) determined by ^1^H NMR, ^13^C NMR and MS data.

### Chemistry

#### General Procedure for Preparation of Compounds **2**–**13**

The DCC (1.2 equiv.) was added to the solution of PD (0.2 mmol), DMAP (0.2 equiv.), and appropriate carboxylic acid (1.2 equiv.) in anhydrous CH_2_Cl_2_ (8 mL) at 0 ºC. The resulting mixture was stirred at room temperature until the starting material was vanished by TLC check. The reaction mixture was filtered and washed with CH_2_Cl_2_ (10 mL × 2). Then, the CH_2_Cl_2_ solution was washed with 5 % HCl (30 mL × 3), saturated NaHCO_3_ (30 mL × 3) and saturated NaCl (30 mL × 3), respectively. Subsequently, the organic layer was dried over anhydrous Na_2_SO_4_ and concentrated to dryness under reduced pressure. The residue was purified by column chromatography over the silica gel to yield the target compound.

##### 3-*O*-Cyclopentanecarbonyl panaxadiol (**2**)

White amorphous powder, yield 74.5 % after chromatography with acetone-petroleum ether (3:97, *v*/*v*); ^1^H NMR (CDCl_3_) *δ* 4.42 (1H, dd, *J* = 11.1, 5.1 Hz, H-3), 3.53 (1H,td, *J* = 10.4, 5.2 Hz, H-12), 2.88 (1H, m, H-2′), 1.14 (6H, overlapped), 1.26 (3H, s, H-26), 1.21 (3H, s, H-28), 1.17 (3H, s, H-27), 1.06 (3H, s, H-21), 0.90 (3H, s, H-30), 0.88 (3H, overlapped, H-29), 0.85 (3H, s, H-19), 0.84 (3H, s, H-18). ^13^C NMR (CDCl_3_) *δ* 176.4 (CO, C-1′), 80.0 (CH, C-3), 76.6 (C, C-25), 73.0 (C, C-20), 69.8 (CH, C-12), 55.9 (CH, C-5), 54.7 (CH, C-17), 51.2 (C, C-14), 49.8 (CH, C-9), 49.1 (CH, C-13), 44.3 (CH, C-2′), 39.8 (C, C-8), 38.5 (CH_2_, C-1), 38.0 (C, C-4), 37.1 (C, C-10), 36.4 (CH_2_, C-24), 35.7 (CH_2_, C-22), 34.8 (CH_2_, C-7), 33.0 (CH_3_, C-26), 31.1 (CH_2_, C-15), 30.5 (CH_2_, C-11), 30.1 (CH_2_, C-3′), 29.8 (CH_2_, C-6′), 28.0 (CH_3_, C-28), 27.1 (CH_3_, C-27), 25.7 (CH_2_, C-4′), 25.6 (CH_2_, C-5′), 25.1 (CH_2_, C-2), 23.7 (CH_2_, C-16), 19.4 (CH_3_, C-21), 18.1 (CH_2_, C-6), 17.0 (CH_3_, C-30), 16.5 (CH_3_, C-29), 16.2 (CH_2_, C-23), 16.1 (CH_3_, C-19), 15.6 (CH_3_, C-18). ESIMS: *m/z* 557 [M+H]^+^, HRESIMS: calcd for C_36_H_61_O_4_ [M+H]^+^ 557.4536, found 557.4564.

##### 3-*O*-(2′-Furoyl) panaxadiol (**3**)

White amorphous powder, yield 62.2 % after chromatography with acetone-petroleum ether (5:95); ^1^H NMR (CDCl_3_) *δ* 7.55 (1H, m, H-4′), 7.11(1H, m, H-2′), 6.48 (1H, m, H-3′), 4.69 (1H, dd, *J* = 10.0, 5.5 Hz, H-3), 3.53 (1H,td, *J* = 10.2, 5.2 Hz, H-12), 1.25 (3H, s, H-27), 1.20 (3H, s, H-26), 1.17 (3H, s, H-21), 0.97 (3H, s, H-18), 0.94 (3H, s, H-28), 0.92 (3H, s, H-29), 0.89 (3H, s, H-19), 0.87 (3H, s, H-30). ^13^C NMR (CDCl_3_) *δ* 158.6 (CO, C-1′), 146.1 (CH, C-5′), 145.1 (CH, C-2′), 117.3 (CH, C-3′), 111.6 (CH, C-4′), 81.2 (CH, C-3), 76.6 (C, C-25), 73.0 (C, C-20), 69.8 (CH, C-12), 55.9 (CH, C-5), 54.7 (CH, C-17), 51.1 (C, C-14), 49.8 (CH, C-9), 49.1 (CH, C-13), 39.6 (C, C-8), 38.5 (CH_2_, C-1), 38.1 (C, C-4), 37.0 (C, C-10), 36.4 (CH_2_, C-24), 35.7 (CH_2_, C-22), 34.7 (CH_2_, C-7), 33.0 (CH_3_, C-26), 31.1 (CH_2_, C-15), 30.5 (CH_2_, C-11), 28.0 (CH_3_, C-28), 27.1 (CH_3_, C-27), 25.1 (CH_2_, C-2), 23.8 (CH_2_, C-16), 19.4 (CH_3_, C-21), 18.1 (CH_2_, C-6), 17.0 (CH_3_, C-30), 16.5 (CH_3_, C-29), 16.2 (CH_2_, C-23), 16.1 (CH_3_, C-19), 15.6 (CH_3_, C-18). ESIMS: *m/z* 555 [M+H]^+^, HRESIMS: calcd for C_35_H_55_O_5_ [M+H]^+^ 555.4044, found 555.4068.

##### 3-*O*-(2′-Thenoyl) panaxadiol (**4**)

White amorphous powder yield 65.8 % after chromatography with acetone-petroleum ether (5:95); ^1^H NMR (CDCl_3_) *δ* 7.78 (1H, dd, *J* = 3.6, 0.9 Hz, H-4′), 7.53 (1H, dd, *J* = 4.9, 0.9 Hz, H-2′), 7.09 (1H, m, H-3′), 4.65 (1H, dd, *J* = 10.9, 4.8 Hz, H-3), 3.55 (1H,td, *J* = 10.4, 5.2 Hz, H-12), 1.26 (3H, s, H-27), 1.21 (3H, s, H-26), 1.18 (3H, s, H-21), 0.98 (3H, s, H-18), 0.96 (3H, s, H-28), 0.93 (3H, s, H-29), 0.91 (3H, s, H-19), 0.88 (3H, s, H-30). ^13^C NMR (CDCl_3_) *δ* 162.0 (CO, C-1′), 134.6 (C, C-2′), 133.0 (CH, C-3′), 132.0 (CH, C-4′), 127.6 (CH, C-4′), 81.8 (CH, C-3), 76.6 (C, C-25), 73.1 (C, C-20), 69.9 (CH, C-12), 55.9 (CH, C-5), 54.7 (CH, C-17), 51.2 (C, C-14), 49.8 (CH, C-9), 49.1 (CH, C-13), 39.8 (C, C-8), 38.5 (CH_2_, C-1), 38.1 (C, C-4), 37.0 (C, C-10), 36.4 (CH_2_, C-24), 35.7 (CH_2_, C-22), 34.7 (CH_2_, C-7), 33.0 (CH_3_, C-26), 31.1 (CH_2_, C-15), 30.5 (CH_2_, C-11), 28.1 (CH_3_, C-28), 27.1 (CH_3_, C-27), 25.1 (CH_2_, C-2), 23.8 (CH_2_, C-16), 19.4 (CH_3_, C-21), 18.2 (CH_2_, C-6), 17.0 (CH_3_, C-30), 16.6 (CH_3_, C-29), 16.2 (CH_2_, C-23), 16.1 (CH_3_, C-19), 15.6 (CH_3_, C-18). ESIMS: *m/z* 571 [M+H]^+^, HRESIMS: calcd for C_35_H_55_O_4_S [M+H]^+^ 571.3816, found 571.3785.

##### 3-*O*-(3′-Methyl)-thenoyl panaxadiol (**5**)

White amorphous powder yield 70.2 % after chromatography with acetone-petroleum ether (5:95); ^1^H NMR (CDCl_3_) *δ* 7.37 (1H, d, *J* = 5.0 Hz, H-5′), 6.91 (1H, d, *J* = 5.0 Hz, H-4′), 4.65 (1H, dd, *J* = 11.3, 4.7 Hz, H-3), 3.56 (td, *J* = 10.4, 5.2 Hz, H-12), 2.56 (3H, s, H-6′), 1.28 (3H, s, H-27), 1.23 (3H, s, H-26), 1.19 (3H, s, H-21), 1.00 (3H, s, H-18), 0.97 (3H, s,H-28), 0.95 (3H, s, H-19), 0.93 (3H, s, H-29), 0.90 (3H, s, H-30). ^13^C NMR (CDCl_3_) *δ* 162.7 (CO, C-1′), 145.6 (C, C-2′), 131.7 (CH, C-5′), 129.7 (CH, C-4′), 127.6 (C, C-3′), 81.5 (CH, C-3), 77.3 (C, C-25), 73.1 (C, C-20), 69.8 (CH, C-12), 55.9 (CH, C-5), 54.7 (CH, C-17), 51.2 (C, C-14), 49.8 (CH, C-9), 49.1 (CH, C-13), 39.8 (C, C-8), 38.5 (C, C-4), 38.1 (CH_2_, C-1), 37.0 (C, C-10), 36.4 (CH_2_, C-24), 35.7 (CH_2_, C-22), 34.8 (CH_2_, C-7), 33.0 (CH_3_, C-26), 31.1 (CH_2_, C-15), 30.5 (CH_2_, C-11), 28.1 (CH_3_, C-28), 27.1 (CH_2_, C-2), 25.1 (CH_3_, C-27), 23.9 (CH_2_, C-16), 19.4 (CH_3_, C-21), 18.2 (CH_2_, C-6), 17.0 (CH_3_, C-30), 16.8 (CH_3_, C-29), 16.2 (CH_2_, C-23), 16.1 (CH_3_, C-19), 15.9 (CH_3_, C-18), 15.6 (CH_3_, C-6′). ESIMS: *m/z* 585 [M+H]^+^, HRESIMS: calcd for C_36_H_57_O_4_S [M+H]^+^ 585.3972, found 585.3938.

##### 3-*O*-(3′-Chloro)-thenoyl panaxadiol (**6**)

White amorphous powder yield 70.2 % after chromatography with acetone-petroleum ether (5:95); ^1^H NMR (CDCl_3_) *δ* 7.43 (1H, d, *J* = 5.3 Hz, H-5′), 6.97 (1H, d, *J* = 5.3 Hz, H-4′), 4.65 (1H, dd, *J* = 11.1, 4.4 Hz, H-3), 3.51 (td, *J* = 10.3, 5.1 Hz, H-12), 1.23 (3H, s, H-27), 1.19 (3H, s, H-26), 1.14 (3H, s, H-21), 0.96 (3H, s, H-18), 0.95 (3H, s,H-28), 0.91 (3H, s, H-19), 0.90 (3H, s, H-29), 0.87 (3H, s, H-30). ^13^C NMR (CDCl_3_) *δ* 160.5 (CO, C-1′), 130.9 (C, C-2′), 130.2 (CH, C-5′), 130.1 (CH, C-4′), 126.7 (C, C-3′), 82.5 (CH, C-3), 76.6 (C, C-25), 73.0 (C, C-20), 69.8 (CH, C-12), 55.9 (CH, C-5), 54.6 (CH, C-17), 51.1 (C, C-14), 49.7 (CH, C-9), 49.1 (CH, C-13), 39.7 (C, C-8), 38.5 (CH_2_, C-1), 38.1 (C, C-4), 37.0 (C, C-10), 36.4 (CH_2_, C-24), 35.7 (CH_2_, C-22), 34.7 (CH_2_, C-7), 33.0 (CH_3_, C-26), 31.1 (CH_2_, C-15), 30.5 (CH_2_, C-11), 28.1 (CH_3_, C-28), 27.1 (CH_3_, C-27), 25.1 (CH_2_, C-2), 23.7 (CH_2_, C-16), 19.4 (CH_3_, C-21), 18.2 (CH_2_, C-6), 17.0 (CH_3_, C-30), 16.7 (CH_2_, C-23), 16.2 (CH_3_, C-29), 16.1 (CH_3_, C-19), 15.6 (CH_3_, C-18). ESIMS: *m/z* 605 [M+H]^+^, HRESIMS: calcd for C_35_H_53_O_4_SCl [M+H]^+^ 605.3426, found 605.3384.

##### 3-*O*-Benzothiophene-2-carboxyl panaxadiol (**7**)

White amorphous powder, yield 87 % after chromatography with acetone-petroleum ether (5:95); ^1^H NMR (CDCl_3_) *δ* 8.03 (1H, s, H-3′), 7.87 (2H, m, H-5′ and H-8′), 7.42 (2H, m, H-6′ and H-7′), 4.72 (1H, m, H-3), 3.55 (1H, td, *J* = 10.3, 5.2 Hz, H-12), 1.28 (6H, s, H-26), 1.23 (6H, s, H-28) 1.19 (3H, s, H-27), 1.02 (3H, s, H-21), 1.01 (3H, s, H-18), 0.96 (3H, H-29), 0.95 (3H, H-19), 0.91 (3H, H-30). ^13^C NMR (CDCl_3_) *δ* 162.5 (CO, C-1′), 142.1 (C, C-9′), 138.7 (C, C-2′), 133.4 (C, C-4′), 130.0 (CH, C-7′), 126.7 (CH, C-6′), 125.4 (CH, C-5′), 124.8 (CH, C-8′), 122.7 (CH, C-3′), 82.3 (CH, C-3), 76.6 (C, C-25), 73.1 (C, C-20), 69.8 (CH, C-12), 55.9 (CH, C-5), 54.7 (CH, C-17), 51.2 (C, C-14), 49.8 (CH, C-9), 49.1 (CH, C-13), 39.8 (C, C-8), 38.5 (CH_2_, C-1), 38.2 (C, C-4), 37.0 (C, C-10), 36.4 (CH_2_, C-24), 35.7 (CH_2_, C-22), 34.8 (CH_2_, C-7), 33.0 (CH_3_, C-26), 31.1 (CH_2_, C-15), 30.5 (CH_2_, C-11), 28.1 (CH_3_, C-28), 27.1 (CH_3_, C-27), 25.1 (CH_2_, C-2), 23.7 (CH_2_, C-16), 19.4 (CH_3_, C-21), 18.2 (CH_2_, C-6), 17.0 (CH_3_, C-30), 16.6 (CH_2_, C-23), 16.2 (CH_3_, C-19), 16.2 (CH_3_, C-29), 15.6 (CH_3_, C-18). ESIMS: *m/z* 621 [M+H]^+^, HRESIMS: calcd for C_39_H_57_O_4_S [M+H]^+^ 621.3972, found 621.3941.

##### 3-*O*-Benzoyl panaxadiol (**8**)

White amorphous powder, yield 68.3 % after chromatography with acetone-petroleum ether (2:98); ^1^H NMR (CDCl_3_) *δ* 8.04 (2H, d, *J* = 7.2 Hz, H-2′), 7.54 (1H, t, *J* = 7.4 Hz, H-4′), 7.43 (2H, t, *J* = 7.6 Hz, H-3′), 4.72 (1H, dd, *J* = 11.2, 4.7 Hz, H-3), 3.55 (2H, m, H-12), 2.19 (2H, t), 1.26 (3H, s, H-27), 1.22 (3H, s, H-26), 1.18 (3H, s, H-21), 1.01 (3H, s, H-28), 1.00 (3H, s, H-29), 0.95 (3H, s, H-18), 0.92 (3H, s, H-19), 0.90 (3H, s, H-30). ^13^C NMR (CDCl_3_) *δ* 166.2 (CO, C-1′), 132.6 (CH, C-5′), 130.9 (C, C-2′), 129.5 (CH, C-3′, C-7′), 128.3 (CH, C-4′, CH, C-6′), 81.5 (CH, C-3), 76.6 (C, C-25), 73.1 (C, C-20), 69.8 (CH, C-12), 56.0 (CH, C-5), 54.7 (CH, C-17), 51.2 (C, C-14), 49.8 (CH, C-9), 49.1 (CH, C-13), 39.8 (C, C-8), 38.5 (CH_2_, C-1), 38.2 (C, C-4), 37.1 (C, C-10), 36.4 (CH_2_, C-24), 35.7 (CH_2_, C-22), 34.8 (CH_2_, C-7), 33.0 (CH_3_, C-26), 31.1 (CH_2_, C-15), 30.5 (CH_2_, C-11), 28.1 (CH_3_, C-28), 27.1 (CH_3_, C-27), 25.1 (CH_2_, C-2), 23.7 (CH_2_, C-16), 19.4 (CH_3_, C-21), 18.2 (CH_2_, C-6), 17.0 (CH_3_, C-30), 16.7 (CH_3_, C-29), 16.2 (CH_3_, C-19), 16.2 (CH_2_, C-23), 15.6 (CH_3_, C-18). ESIMS(+): *m*/*z* 565 [M+H]^+^, HRESIMS: calcd for C_37_H_57_O_4_ [M+H]^+^ 565.4251, found 565.4232.

##### 3-*O*-Salicyloyl panaxadiol (**9**)

White amorphous powder, yield 59.0 % after chromatography with formic acid-acetone-petroleum ether (0.5:5:95);^1^H NMR (CD_3_OD) *δ* 7.83 (1H, d, *J* = 7.7 Hz, H-7′), 7.44 (1H, d, *J* = 7.5 Hz, H-4′), 6.89 (1H, d, *J* = 7.7 Hz, H-6′), 6.87 (1H, d, *J* = 7.5 Hz, H-5′), 4.75 (1H, dd, *J* = 10.1, 5.8 Hz, H-3), 3.55 (td, *J* = 10.2, 5.0 Hz, H-12), 1.27 (3H, s, H-27), 1.22 (3H, s, H-26), 1.18 (3H, s, H-21), 1.01 (3H, s, H-18), 1.00 (3H, s,H-28), 0.95 (3H, s, H-19), 0.92 (3H, s, H-29), 0.90 (3H, s, H-30). ^13^C NMR (CD_3_OD) *δ* 169.9 (CO, C-1′), 161.6 (C, C-3′), 135.4 (CH, C-5′), 129.7 (C-7′), 119.0 (CH, C-6′), 117.5 (CH, C-4′), 113.0 (C, C-2′), 82.2 (CH, C-3), 76.6 (C, C-25), 73.1 (C, C-20), 69.8 (CH, C-12), 55.9 (CH, C-5), 54.6 (CH, C-17), 51.1 (C, C-14), 49.8 (CH, C-9), 49.1 (CH, C-13), 39.7 (C, C-8), 38.5 (CH_2_, C-1), 38.2 (C, C-4), 37.0 (C, C-10), 36.4 (CH_2_, C-24), 35.7 (CH_2_, C-22), 34.7 (CH_2_, C-7), 33.0 (CH_3_, C-26), 31.1 (CH_2_, C-15), 30.5 (CH_2_, C-11), 28.1 (CH_3_, C-28), 27.1 (CH_3_, C-27), 25.1 (CH_2_, C-2), 23.7 (CH_2_, C-16), 19.3 (CH_3_, C-21), 18.1 (CH_2_, C-6), 17.0 (CH_3_, C-30), 16.7 (CH_2_, C-23), 16.2 (CH_3_, C-29), 16.1 (CH_3_, C-19), 15.6 (CH_3_, C-18). ESIMS: *m/z* 581 [M+H]^+^, HRESIMS: calcd for C_37_H_57_O_5_ [M+H]^+^ 581.4201, found 581.4158.

##### 3-*O*-Galloyl panaxadiol (**10**)

White amorphous powder, yield 15.1 % after chromatography with formic acid-acetone-petroleum ether (0.5:10:90); ^1^H NMR (CDCl_3_) *δ* 7.05 (2H, s, H-3′, H-7′),4.04 (1H, t, *J* = 8.4 Hz, H-3), 3.62 (td, *J* = 10.3, 5.1 Hz, H-12), 1.17 (3H, s, H-27), 1.06 (3H, s, H-26), 0.92 (3H, s, H-21), 0.89 (6H, overlapped, H-18, H-28), 0.74 (3H, s, H-19), 0.70 (6H, overlapped, H-29, H-30). ^13^C NMR (CDCl_3_) *δ* 166.7 (CO, C-1′), 144.2 (C, C-4′, CH, C-6′), 136.8 (C, C-5′), 122.6 (C, C-2′), 109.4 (CH, C-3′, C-7′), 78.7 (CH, C-3), 77.2 (C, C-25), 75.2 (CH, C-12), 75.2 (C, C-20), 70.7 (CH, C-5), 55.7 (CH, C-17), 53.8 (C, C-14), 51.9 (CH, C-9), 49.5 (CH, C-13), 45.0 (C, C-8), 39.4 (C, C-4), 38.8 (C, C-10), 38.6 (CH_2_, C-1), 37.1 (CH_2_, C-24), 34.7 (CH_2_, C-22), 34.2 (CH_2_, C-7), 32.9 (CH_3_, C-26), 30.5 (CH_2_, C-15), 29.6 (CH_2_, C-11), 27.9 (CH_3_, C-28), 27.6 (CH_3_, C-27), 26.9 (CH_2_, C-2), 26.7 (CH_3_, C-21), 25.6 (CH_2_, C-6), 17.9 (CH_3_, C-30), 16.4 (CH_2_, C-23), 15.7 (CH_3_, C-29), 15.6 (CH_3_, C-19), 15.3 (CH_3_, C-18). ESIMS: *m/z* 613 [M+H]^+^, HRESIMS: calcd for C_37_H_57_O_7_ [M+H]^+^ 613.4099, found 613.4055.

##### 3-*O*-Nicotinoyl panaxadiol (**11**)

White amorphous powder, yield 84.0 % after chromatography with acetone-petroleum ether (12.5:87.5); ^1^H NMR (CDCl_3_) *δ* 9.21 (1H, d, *J* = 1.5 Hz, H-2′), 8.75 (1H, dd, *J* = 4.8, 1.5 Hz, H-3′), 8.27 (1H, m, H-5′), 7.37 (1H, dd, *J* = 7.9, 4.9 Hz, H-4′), 4.74 (1H, dd, *J* = 10.6, 5.3 Hz, H-3), 3.53 (1H,td, *J* = 10.3, 5.2 Hz, H-12), 2.19 (2H, t), 1.25 (3H, s, H-27), 1.20 (3H, s, H-26), 1.17 (3H, s, H-21), 0.99 (3H, s, H-28), 0.98 (3H, s, H-29), 0.94 (3H, s, H-18), 0.91 (3H, s, H-19), 0.88 (3H, s, H-30). ^13^C NMR (CDCl_3_) *δ* 164.9 (CO, C-1′), 153.2 (CH, C-4′), 150.8 (CH, C-3′), 137.0 (CH, C-6′), 126.7 (C, C-2′), 123.2 (CH, C-5′).82.2 (CH, C-3), 76.6 (C, C-25), 73.1 (C, C-20), 69.8 (CH, C-12), 56.0 (CH, C-5), 54.7 (CH, C-17), 51.2 (C, C-14), 49.8 (CH, C-9), 49.1 (CH, C-13), 39.8 (C, C-8), 38.5 (CH_2_, C-1), 38.2 (C, C-4), 37.1 (C, C-10), 36.4 (CH_2_, C-24), 35.7 (CH_2_, C-22), 34.8 (CH_2_, C-7), 33.0 (CH_3_, C-26), 31.1 (CH_2_, C-15), 30.5 (CH_2_, C-11), 28.1 (CH_3_, C-28), 27.1 (CH_3_, C-27), 25.1 (CH_2_, C-2), 23.7 (CH_2_, C-16), 19.4 (CH_3_, C-21), 18.2 (CH_2_, C-6), 17.0 (CH_3_, C-30), 16.7 (CH_3_, C-29), 16.2 (CH_2_, C-23), 16.1 (CH_3_, C-19), 15.6 (CH_3_, C-18). ESIMS(+): *m*/*z* 566 [M+H]^+^, HRESIMS: calcd for C_36_H_56_NO_4_ [M+H]^+^ 566.4204, found 566.4183.

##### 3-*O*-Valeryl panaxadiol (**12**)

White amorphous powder, yield 85.0 % after chromatography with acetone-petroleum ether (2:98); ^1^H NMR (CDCl_3_) *δ* 4.48 (1H, dd, *J* = 11.1, 5.3 Hz, H-3), 3.53 (1H, td, *J* = 10.2, 5.2 Hz, H-12), 2.19 (2H, t, H-2′), 1.26 (3H, s, H-27), 1.21 (3H, s, H-26), 1.17 (3H, s, H-21), 0.97 (3H, s, H-28), 0.93 (3H, s, H-29), 0.89 (3H, s, H-18), 0.85 (3H, s, H-19), 0.84 (3H, s, H-30). ^13^C NMR (CDCl_3_) *δ* 173.6 (CO, C-1′), 80.5 (CH, C-3), 76.6 (C, C-25), 73.0 (C, C-20), 69.8 (CH, C-12), 55.9 (CH, C-5), 54.7 (CH, C-17), 51.2 (C, C-14), 49.8 (CH, C-9), 49.1 (CH, C-13), 39.7 (C, C-8), 38.5 (CH_2_, C-1), 37.8 (C, C-4), 37.0 (C, C-10), 36.4 (CH_2_, C-24), 35.7 (CH_2_, C-22), 34.8 (CH_2_, C-7), 34.5 (CH_2_, C-2′), 33.0 (CH_3_, C-26), 31.1 (CH_2_, C-15), 30.5 (CH_2_, C-11), 27.9 (CH_3_, C-28), 27.2 (CH_2_, C-3′), 27.1 (CH_3_, C-27), 25.1 (CH_2_, C-2), 23.7 (CH_2_, C-16), 22.3 (CH_2_, C-4′), 19.4 (CH_3_, C-21), 18.1 (CH_2_, C-6), 17.0 (CH_3_, C-30), 16.5 (CH_3_, C-29), 16.2 (CH_2_, C-23), 16.1 (CH_3_, C-19), 15.6 (CH_3_, C-18), 13.7 (CH_3_, C-5′). ESIMS(+): *m*/*z* 545 [M+H]^+^, HRESIMS: calcd for C_35_H_61_O_4_ [M+H]^+^ 545.4564, found 545.4564.

##### 3-*O*-2′-Ethyoxyl-acetyl panaxadiol (**13**)

White amorphous powder, yield 74.1 % after chromatography with acetone-petroleum ether (5:95); ^1^H NMR (CDCl_3_) *δ* 4.58 (1H, m, H-3), 4.05 (2H, q), 3.58 (2H, m), 3.53 (1H,td, *J* = 10.3, 5.2 Hz, H-12), 1.24 (6H, overlapped), 1.21 (3H, s, H-26), 1.17 (3H, s, H-21), 0.97 (3H, s, H-28), 0.89 (3H, s, H-29), 0.87(3H, s, H-18), 0.84 (6H, s). ^13^C NMR (CDCl_3_) *δ* 170.4 (CO, C-1′), 81.4 (CH, C-3), 76.6 (C, C-25), 73.1 (C, C-20), 69.8 (CH, C-12), 68.2 (CH_2_, C-2′), 67.1 (CH_2_, C-3′), 55.8 (CH, C-5), 54.7 (CH, C-17), 51.2 (C, C-14), 49.8 (CH, C-9), 49.1 (CH, C-13), 39.7 (C, C-8), 38.5 (CH_2_, C-1), 37.9 (C, C-4), 37.0 (C, C-10), 36.4 (CH_2_, C-24), 35.7 (CH_2_, C-22), 34.7 (CH_2_, C-7), 33.0 (CH_3_, C-26), 31.1 (CH_2_, C-15), 30.5 (CH_2_, C-11), 28.0 (CH_3_, C-28), 27.1 (CH_3_, C-27), 25.1 (CH_2_, C-2), 23.7 (CH_2_, C-16), 19.4 (CH_3_, C-21), 18.1 (CH_2_, C-6), 17.0 (CH_3_, C-30), 16.4 (CH_3_, C-29), 16.2 (CH_2_, C-23), 16.1 (CH_3_, C-19), 15.6 (CH_3_, C-18), 15.0 (CH_3_, C-4′). ESIMS(+): *m*/*z* 547 [M+H]^+^, HRESIMS: calcd for C_34_H_59_O_5_ [M+H]^+^ 547.4357, found 547.4336.

#### General Procedure for Preparation of Compounds **1** and **14**–**19**

A solution of panaxadiol (0.5 mmol), the corresponding anhydride (3 equiv.) in anhydrous pyridine (6 mL) was added DMAP (0.3 equiv.) and stirred at 90 ºC for 5 h. The cooling reaction mixture was diluted with ice water (30 mL), extracted with ethyl acetate (30 mL × 3). The ethyl acetate mixture was washed with 5 % HCl (30 mL × 3) and saturated NaCl (30 mL × 3). The ethyl acetate layer was dried over anhydrous Na_2_SO_4_ and concentrated to dryness under reduced pressure. The crude products were purified by silica gel column chromatography.

##### 3-*O*-Acetly-panaxadiol (**1**)

White amorphous powder yield 93 % after chromatography with acetone-petroleum ether (10:90); ^1^H NMR (CDCl_3_) *δ* 4.47 (1H, m, H-3), 3.52 (1H, td, *J* = 10.4, 5.2 Hz, H-12), 2.04 (3H, s, COCH_3_), 1.27 (3H, s, H-26), 1.24 (3H, s, H-28), 1.17 (3H, s, H-27), 0.97 (3H, s, H-21), 0.89 (3H, s, H-30), 0.87 (3H, t, H-29), 0.84 (6H, overlapped). ^13^C NMR (CDCl_3_) *δ* 171.0 (CO, C-1′), 80.8 (CH, C-3), 76.6 (C, C-25), 73.1 (C, C-20), 69.8 (CH, C-12), 55.9 (CH, C-5), 54.7 (CH, C-17), 51.2 (C, C-14), 49.8 (CH, C-9), 49.1 (CH, C-13), 39.7 (C, C-8), 38.5 (CH_2_, C-1), 37.4 (C, C-4), 36.8 (C, C-10), 36.4 (CH_2_, C-24), 35.7 (CH_2_, C-22), 34.8 (CH_2_, C-7), 33.0 (CH_3_, C-26), 31.1 (CH_2_, C-15), 30.6 (CH_2_, C-11), 27.9 (CH_3_, C-28), 27.1 (CH_3_, C-27), 25.1 (CH_2_, C-2), 23.7 (CH_2_, C-16), 21.3 (CH_3_, C-2′), 19.4 (CH_3_, C-21), 18.1 (CH_2_, C-6), 17.0 (CH_3_, C-30), 16.4 (CH_3_, C-29), 16.2 (CH_2_, C-23), 16.1 (CH_3_, C-19), 15.6 (CH_3_, C-18). ESIMS: *m/z* 503 [M+H]^+^, HRESIMS: calcd for C_32_H_53_O_4_ [M+H]^+^ 503.4095, found 503.4062.

##### 3-*O*-Succinyl panaxadiol (**14**)

White amorphous powder, yield 69.1 % after chromatography with formic acid-acetone-petroleum ether (0.4:12.5:87.5); ^1^H NMR (CDCl_3_) *δ* 4.50 (1H, dd, *J* = 10.1, 6.4 Hz, H-3), 3.58 (1H, td, *J* = 10.3, 5.2 Hz, H-12), 2.64 (4H, m, H-2′, H-3′), 1.26 (3H, s, H-27), 1.21 (3H, s, H-26), 1.18 (3H, s, H-21), 0.98 (3H, s, H-18), 0.89 (3H, s, H-28), 0.87(3H, s, H-19), 0.84 (6H, overlapped). ^13^C NMR (CDCl_3_) *δ* 176.2 (COOH, C-4′), 171.8 (CO, C-1′), 81.3 (CH, C-3), 76.7 (C, C-25), 73.4 (C, C-20), 70.3 (CH, C-12), 55.9 (CH, C-5), 54.6 (CH, C-17), 51.2 (C, C-14), 49.8 (CH, C-9), 48.8 (CH, C-13), 39.7 (C, C-8), 38.6 (CH_2_, C-1), 37.9 (C, C-4), 37.0 (C, C-10), 36.4 (CH_2_, C-24), 35.7 (CH_2_, C-22), 34.8 (CH_2_, C-7), 32.9 (CH_3_, C-26), 31.1 (CH_2_, C-15), 30.1 (CH_2_, C-11), 29.4 (CH_2_, C-2′), 29.0 (CH_2_, C-3′), 27.9 (CH_3_, C-28), 27.0 (CH_3_, C-27), 25.1 (CH_2_, C-2), 23.8 (CH_2_, C-16), 19.3 (CH_3_, C-21), 18.1 (CH_2_, C-6), 17.0 (CH_3_, C-30), 16.5 (CH_3_, C-29), 16.2 (CH_3_, C-19), 16.2 (CH_2_, C-23), 15.6 (CH_3_, C-18). ESIMS(+): *m*/*z* 561 [M+H]^+^, HRESIMS: calcd for C_34_H_51_O_6_ [M+H]^+^ 561.4121, found 561.4150.

##### 3-*O*-Glutaryl panaxadiol (**15**)

White amorphous powder, yield 73.1 % after chromatography with formic acid-acetone-petroleum ether (0.4:12.5:87.5); ^1^H NMR (CDCl_3_) *δ* 4.47 (1H, dd, *J* = 9.7, 6.9 Hz, H-3), 3.58 (1H, td, *J* = 10.3, 5.1 Hz, H-12), 1.25 (3H, s, H-27), 1.20 (3H, s, H-26), 1.16 (3H, s, H-21), 0.96 (3H, s, H-18), 0.88 (3H, s, H-28), 0.86 (3H, s, H-19), 0.82 (6H, overlapped). ^13^C NMR (CDCl_3_) *δ* 177.3 (COOH, C-4′), 172.7 (CO, C-1′), 80.8 (CH, C-3), 77.3 (C, C-25), 73.4 (C, C-20), 70.3 (CH, C-12), 55.9 (CH, C-5), 54.6 (CH, C-17), 51.2 (C, C-14), 49.7 (CH, C-9), 48.8 (CH, C-13), 39.7 (C, C-8), 38.5 (CH_2_, C-1), 37.8 (C, C-4), 37.0 (C, C-10), 36.4 (CH_2_, C-24), 35.7 (CH_2_, C-22), 34.7 (CH_2_, C-7), 33.7 (CH_2_, C-4′), 33.1 (CH_2_, C-3′), 32.9 (CH_3_, C-26), 31.1 (CH_2_, C-15), 30.0 (CH_2_, C-11), 28.0 (CH_3_, C-28), 27.0 (CH_3_, C-27), 25.1 (CH_2_, C-2), 23.7 (CH_2_, C-16), 20.1 (CH_2_, C-3′), 19.3 (CH_3_, C-21), 18.1 (CH_2_, C-6), 16.9 (CH_3_, C-30), 16.5 (CH_3_, C-29), 16.2 (CH_3_, C-19), 16.1 (CH_2_, C-23), 15.5 (CH_3_, C-18). ESIMS(+): *m*/*z* 575 [M+H]^+^, HRESIMS: calcd for C_35_H_51_O_6_ [M+H]^+^ 575.4306, found 575.4281.

##### 3-*O*-Diglycolyl panaxadiol (**16**)

White amorphous powder, yield 47.3 % after chromatography with formic acid-ethyl acetate-petroleum ether (0.40:40:60);^1^H NMR (CDCl_3_) *δ* 4.53 (1H, dd, *J* = 9.9, 6.4 Hz, H-3), 4.17 (4H, s, H-2′, H-3′), 3.54 (1H,td, *J* = 10.2, 5.1 Hz, H-12), 1.20 (3H, s, H-27), 1.14 (3H, s, H-26), 1.12 (3H, s, H-21), 0.91 (3H, s, H-18), 0.83 (3H, s, H-28), 0.81 (3H, s, H-19), 0.78 (3H, s, H-30), 0.77 (3H, s, H-29). ^13^C NMR (CDCl_3_) *δ* 171.8 (COOH, C-4′), 170.0 (CO, C-1′), 81.9 (CH, C-3), 76.8 (C, C-20), 73.5 (C, C-25), 70.4 (CH, C-12), 68.2 (2 × CH_2_, C-2′, C-3′), 55.8 (CH, C-13), 54.5 (CH, C-5), 51.2 (C, C-14), 49.7 (CH, C-9), 48.6 (CH, C-17), 39.7 (C, C-4), 38.3 (CH_2_, C-11), 37.9 (C, C-8), 36.9 (C, C-10), 36.3 (CH_2_, C-1), 35.6 (CH_2_, C-24), 34.6 (CH_2_, C-22), 32.8 (CH_3_, C-26), 31.1 (CH_2_, C-7), 29.7 (CH_2_, C-15), 28.0 (CH_3_, C-28), 26.9 (CH_3_, C-27), 25.0 (CH_2_, C-2), 23.6 (CH_2_, C-16), 19.2 (CH_3_, C-21), 18.0 (CH_2_, C-6), 16.9 (CH_3_, C-30), 16.4 (CH_3_, C-29), 16.1 (CH_3_, C-19), 16.1 (CH_2_, C-23), 15.5 (CH_3_, C-18). ESIMS(+): *m*/*z* 577 [M+H]^+^, HRESIMS: calcd for C_34_H_51_O_7_ [M+H]^+^ 577.4099, found 577.4064.

##### 3-*O*-(3′-Methyl) diglutaryl panaxadiol (**17**)

White amorphous powder, yield 35.1 % after chromatography with formic acid-ethyl acetate-petroleum ether (0.40:40:60); ^1^H NMR (CDCl_3_) *δ* 4.42 (1H, t, *J* = 8.2 Hz, H-3), 3.46 (td, *J* = 10.4, 5.1 Hz, H-12), 1.18 (3H, s, H-27), 1.13 (3H, s, H-26), 1.09 (3H, s, H-21), 0.96 (3H, t, *J* = 5.8 Hz, H-6′), 0.90 (3H, s, H-18), 0.83 (3H, s,H-28), 0.80 (3H, s, H-19), 0.78 (3H, s, H-29), 0.77 (3H, s, H-30). ^13^C NMR (CDCl_3_) *δ* 174.9 (COOH, C-5′), 172.6 (CO, C-1′), 81.0 (CH, C-3), 76.4 (C, C-25), 73.2 (C, C-20), 70.0 (CH, C-12), 55.8 (CH, C-5), 54.5 (CH, C-17), 51.1 (C, C-14), 49.6 (CH, C-9), 48.8 (CH, C-13), 48.7 (CH, C-3′), 41.3 (CH_2_, C-2′), 40.6 (CH_2_, C-4′), 39.6 (C, C-8), 38.4 (CH_2_, C-1), 37.7 (C, C-4), 36.9 (C, C-10), 36.2 (CH_2_, C-24), 35.6 (CH_2_, C-22), 34.6 (CH_2_, C-7), 32.8 (CH_3_, C-26), 31.0 (CH_2_, C-15), 30.0 (CH_2_, C-11), 27.9 (CH_3_, C-28), 26.9 (CH_3_, C-27), 25.0 (CH_2_, C-2), 23.5 (CH_2_, C-16), 19.6 (CH_3_, C-6′), 19.2 (CH_3_, C-21), 18.0 (CH_2_, C-6), 16.8 (CH_3_, C-30), 16.4 (CH_3_, C-29), 16.1 (CH_2_, C-23), 16.0 (CH_3_, C-19), 15.4 (CH_3_, C-18). ESIMS(+): *m*/*z* 589 [M+H]^+^, HRESIMS: calcd for C_36_H_61_O_6_ [M+H]^+^ 589.4463, found 575.4435.

##### 3-*O*-(3′,3′-Dimethyl) glutaryl panaxadiol (**18**)

White amorphous powder, yield 42.6 % after chromatography with ethyl acetate-petroleum ether (35:65); ^1^H NMR (CDCl_3_) *δ*: 4.42 (1H, dd, *J* = 10.8, 5.5 Hz, H-3), 3.49 (1H, dd, *J* = 10.3, 5.1 Hz, H-12), 1.19 (3H, s, H-27), 1.14 (3H, s, H-26), 1.11 (3H, s, H-21), 1.06 (6H, s, H-28, H-18), 0.90 (3H, s, H-19), 0.82 (3H, s, H-29), 0.80 (3H, s, H-30). ^13^C NMR (CDCl_3_) *δ*: 176.2 (CO, C-5′), 172.2 (CO, C-1′), 81.1 (CH, C-3), 77.2 (C, C-25), 73.2 (C, C-20), 70.2 (CH, C-12), 55.8 (CH, C-5), 54.7 (CH, C-17), 51.2 (C, C-14), 49.8 (CH, C-9), 48.9 (CH, C-13), 45.6 (CH_2_, C-4′), 45.4 (CH_2_, C-2′), 39.8 (C, C-8), 38.5 (C, C-4), 38.4 (CH_2_, C-1), 37.7 (C, C-10), 37.0 (C, C-3′), 36.4 (CH_2_, C-24), 35.7 (CH_2_, C-22), 34.8 (CH_2_, C-7), 32.9 (CH_3_, C-26), 31.1 (CH_2_, C-15), 30.2 (CH_2_, C-11), 28.1 (CH_3_, C-6′), 27.8 (CH_3_, C-28), 27.7 (CH_3_, C-7′), 27.1 (CH_3_, C-27), 25.1 (CH_2_, C-2), 23.8 (CH_2_, C-16), 19.4 (CH_3_, C-21), 18.2 (CH_2_, C-6), 17.0 (CH_3_, C-30), 16.6 (CH_3_, C-29), 16.2 (CH_2_, C-23), 16.2 (CH_3_, C-19), 15.6 (CH_3_, C-18). ESIMS(+): m/z 603 [M+H]^+^, HRESIMS: calcd for C_37_H_63_O_6_ [M+H]^+^ 603.4619, found 603.4619.

##### 3-*O*-(3′,3′-tetramethylene)diglutaryl panaxadiol (**19**)

White amorphous powder, yield 54.1 % after chromatography with ethyl acetate-petroleum ether (35:65); ^1^H NMR (CDCl_3_) *δ*: 4.42 (1H, dd, *J* = 10.8, 5.5 Hz, H-3), 3.49 (1H, dd, *J* = 10.3, 5.1 Hz, H-12), 1.19 (3H, s, H-27), 1.14 (3H, s, H-26), 1.11 (3H, s, H-21), 1.06 (6H, s, H-28, H-18), 0.90 (3H, s, H-19), 0.82 (3H, s, H-29), 0.80 (3H, s, H-30). ^13^C NMR (CDCl_3_) *δ* 176.2 (CO, C-5′), 172.2 (CO, C-1′), 81.1 (CH, C-3), 77.2 (C, C-25), 73.2 (C, C-20), 70.2 (CH, C-12), 55.8 (CH, C-5), 54.7 (CH, C-17), 51.2 (C, C-14), 49.8 (CH, C-9), 48.9 (CH, C-13), 45.6 (CH_2_,, C-4′), 45.4 (CH_2_, C-2′), 39.8 (C, C-8), 38.5 (C, C-4), 38.4 (CH_2_, C-1), 37.7 (C, C-10), 37.0 (C, C-3′), 36.4 (CH_2_, C-24), 35.7 (CH_2_, C-22), 34.8 (CH_2_, C-7), 32.9 (CH_3_, C-26), 31.1 (CH_2_, C-15), 30.2 (CH_2_, C-11), 28.1 (CH_2_, C-6′), 27.8 (CH_3_, C-28), 27.7 (CH_2_, C-7′), 27.1 (CH_3_, C-27), 25.1 (CH_2_, C-2), 23.8 (CH_2_, C-16), 19.4 (CH_3_, C-21), 18.2 (CH_2_, C-6), 17.0 (CH_3_, C-30), 16.6 (CH_3_, C-29), 16.2 (CH_2_, C-23), 16.2 (CH_3_, C-19), 15.6 (CH_3_, C-18). ESIMS(+): *m*/*z* 629 [M+H]^+^, HRESIMS: calcd for C_39_H_65_O_6_ [M+H]^+^ 629.4776, found 629.4778.

#### General Procedure for Preparation of Compounds **20** and **21**

Tow derivatives were obtained with Panaxatriol (0.5 mmol), the anhydride (3 equiv.) and DMAP (0.3 equiv.) in anhydrous pyridine at 90 ºC for 5 h. Then the mixture was treated by the way similar to compounds **20** and **21**.

##### 3, 6-*O*-Diacetyl-panaxatriol (**20**)

White amorphous powder, yield 98.1 % after chromatography with formic acid-ethyl acetate-petroleum ether (10:90); ^1^H NMR (CDCl_3_) *δ* 5.35 (1H, m, H-6), 4.46 (1H, dd, *J* = 11.4, 5.3 Hz, H-3), 3.53 (1H, td, *J* = 10.3, 5.2 Hz, H-12), 2.06 (3H, s, H-2′), 2.04 (3H, s, H-2′′), 1.26 (3H, s, H-27), 1.22 (3H, s, H-26), 1.18 (3H, s, H-21), 1.11 (3H, s, H-18), 1.02 (3H, s, H-28), 1.01 (3H, s, H-19), 0.91 (3H, s, H-29), 0.90 (3H, s, H-30). ^13^C NMR (CDCl_3_) *δ* 171.0 (CO, C-1′), 170.1 (CO, C-1′′), 80.2 (CH, C-3), 76.5 (C, C-20), 73.1 (C, C-25), 70.6 (CH_2_, C-6), 69.6 (CH, C-12), 58.6 (CH, C-5), 54.6 (CH, C-17), 51.0 (C, C-14), 49.2 (CH, C-9), 48.7 (CH, C-13), 42.5 (CH_2_, C-7), 40.7 (C, C-8), 39.2 (C, C-10), 38.3 (CH_2_, C-1), 37.6 (C, C-4), 36.3 (CH_2_, C-24), 35.6 (CH_2_, C-22), 33.0 (CH_3_, C-26), 31.0 (CH_2_, C-15), 30.3 (CH_2_, C-11), 30.2 (CH_3_, C-28), 27.1 (CH_3_, C-27), 25.0 (CH_2_, C-16), 23.2 (CH_2_, C-2), 22.0 (CH_3_, C-2′), 21.3 (CH_3_, C-2′′), 19.3 (CH_3_, C-21), 17.1 (CH_3_, C-19), 16.9 (CH_3_, C-30), 16.8 (CH_3_, C-18), 16.8 (CH_3_, C-29), 16.2 (CH_2_, C-23). ESIMS(+): *m*/*z* 561 [M+H]^+^, HRESIMS: calcd for C_34_H_57_O_6_ [M+H]^+^ 561.4150, found 561.4146.

##### 3, 6-*O*-Disuccinyl panaxatriol (**21**)

White amorphous powder, yield 45.8 % after chromatography with formic acid-ethyl acetate-petroleum ether (0.40:40:60); ^1^H NMR (CDCl_3_) *δ* 5.35 (1H, m, H-6), 4.46 (1H, dd, *J* = 11.1, 5.2 Hz, H-3), 3.53 (1H,td, *J* = 10.3, 5.1 Hz, H-12), 1.23 (3H, s, H-27), 1.17 (3H, s, H-26), 1.14 (3H, s, H-21), 1.08 (3H, s, H-18), 1.00 (3H, s, H-28), 0.98 (3H, s, H-19), 0.87 (3H, s, H-29), 0.86 (3H, s, H-30). ^13^C NMR (CDCl_3_) *δ* 177.1 (COOH, C-4′), 176.9 (COOH, C-4′′), 171.9 (CO, C-1′), 171.6 (CO, C-1′′), 80.7 (CH, C-3), 76.7 (C, C-20), 73.5 (C, C-25), 71.1 (CH, C-12), 70.1 (C- 6), 58.6 (CH,C-5), 54.5 (CH, C-17), 51.0 (C, C-14), 49.2 (CH, C-9), 48.3 (CH, C-13), 42.3 (CH_2_, C-7), 40.7 (C, C-8), 39.2 (C, C-4), 38 (C, C-10), 37.7 (CH_2_, C-1), 36.3 (CH_2_, C-24), 35.6 (CH_2_, C-22), 32.8 (CH_3_, C-26), 30.2 (CH_3_, C-28), 29.8 (CH_2_, C-3′), 29.7 (CH_2_, C-11), 29.4 (CH_2_, C-3′′), 29.0 (CH_2_, C-2′), 28.8 (CH_2_, C-2′′), 27.0 (CH_3_, C-27), 19.2 (CH_3_, C-21), 17.1 (CH_3_, C-18), 16.9 (CH_3_, C-19), 16.7 (CH_3_, C-30), 16.6 (CH_3_, C-29), 16.1 (CH_2_, C-23). ESIMS(+): *m*/*z* 677 [M+H]^+^, HRESIMS: calcd for C_38_H_61_O_10_ [M+H]^+^ 677.4259, found 677.4223.

#### General Procedure for Preparation of Compounds **22**–**25**

These derivatives were synthesized by the Steglich esterification reaction of panaxatriol (0.5 mmol) with the corresponding acid (1.5 equiv.) and DCC (1.5 equiv.) in the presence of DMAP (0.8 equiv.), at the similar treatment process to preparation of compounds **22**–**25**

##### 3,6-*O*-Di(2′-furoyl)-panaxatriol (**22**)

White amorphous powder, yield 78.2 % after chromatography with ethyl acetate-petroleum ether (10:90); ^1^H NMR (CDCl_3_) *δ* 5.58 (1H, t, *J* = 8.8 Hz, H-6), 4.68 (1H, m, H-3), 1.22 (3H, s, H-27), 1.18 (6H, s, H-21, H-18), 1.17 (3H, s, H-26), 1.11 (3H, s, H-28), 1.05 (3H, s, H-19), 1.00 (3H, s, H-29), 0.87 (3H, s, H-30). ^13^C NMR (CDCl_3_) *δ* 158.7 (CO, C-1′), 158.0 (CO, C-1′′), 146.4 (CH, C-5′), 146.3 (CH, C-5′′), 145.0 (C, C-2′), 144.8 (C, C-2′′), 118.1 (CH, C-3′), 117.6 (CH, C-3′′), 111.8 (CH, C-4′), 111.7 (CH, C-4′′), 81.0 (CH, C-3), 76.6 (C, C-20), 73.2 (C, C-25), 71.4 (CH_2_, C-6), 69.7 (CH, C-12), 58.7 (CH, C-5), 54.5 (CH, C-17), 51.0 (C, C-14), 49.2 (CH, C-9), 48.5 (CH, C-13), 42.4 (CH_2_, C-7), 40.8 (C, C-8), 39.3 (C, C-4), 38.1 (CH_2_, C-1), 38.0 (C, C-10), 36.3 (CH_2_, C-24), 35.6 (CH_2_, C-22), 33.7 (CH_2_, C-15), 32.9 (CH_3_, C-26), 31.0 (CH_2_, C-11), 30.4 (CH_3_, C-28), 27.0 (CH_3_, C-27), 25.5 (CH_2_, C-2), 24.8 (CH_2_, C-16), 23.3 (CH_3_, C-21), 19.3 (CH_3_, C-18), 17.1 (CH_3_, C-19), 16.9 (CH_3_, C-30), 16.8 (CH_3_, C-29), 16.1 (CH_2_, C-23). ESIMS(+): *m*/*z* 656 [M+H]^+^, HRESIMS: calcd for C_40_H_57_O_8_ [M+H]^+^ 665.4048, found 665.4002.

##### 3,6-*O*-Di(2′-thenoyl)-panaxatriol (**23**)

White amorphous powder, yield 82.7 % after chromatography with ethyl acetate-petroleum ether (10:90); ^1^H NMR (CDCl_3_) *δ* 5.35 (1H, t, *J* = 8.9 Hz, H-6), 4.93 (1H, m, H-3), 1.23 (3H, s, H-27), 1.17 (3H, s, H-26), 1.05 (6H, s, H-21, H-18), 1.02 (3H, s,H-28), 0.95 (3H, s, H-19), 0.90 (3H, s, H-29), 0.87 (3H, s, H-30). ^13^C NMR (CDCl_3_) *δ* 162.8 (CO, C-1′), 158.0 (CO, C-1′′), 134.5 (C, C-2′), 134.1 (C, C-2′′), 133.3 (CH, C-3′), 133.0 (CH, C-3′′), 132.4 (CH, C-4′), 132.1 (CH, C-4′′), 127.6 (CH, C-5′), 127.5 (CH, C-5′′).81.0 (CH, C-3), 76.4 (C, C-20), 73.0 (C, C-25), 71.5 (CH_2_, C-6), 69.4 (CH, C-12), 58.6 (CH, C-5), 54.4 (CH, C-17), 50.9 (C, C-14), 49.9 (CH, C-9), 49.1 (CH, C-13), 42.3 (CH_2_, C-7), 40.7 (C, C-8), 39.3 (C, C-4), 38.1 (CH_2_, C-1), 37.9 (C, C-10), 36.3 (CH_2_, C-24), 35.5 (CH_2_, C-22), 32.9 (CH_2_, C-15), 32.1 (CH_3_, C-26), 31.0 (CH_2_, C-11), 30.2 (CH_3_, C-28), 27.0 (CH_3_, C-27), 25.2 (CH_2_, C-2), 25.1 (CH_2_, C-16), 23.4 (CH_3_, C-21), 19.2 (CH_3_, C-18), 17.1 (CH_3_, C-19), 16.9 (CH_3_, C-30), 16.8 (CH_3_, C-29), 16.1 (CH_2_, C-23). ESIMS(+): *m*/*z* 697 [M+H]^+^, HRESIMS: calcd for C_40_H_57_O_6_S_2_ [M+H]^+^ 697.3591, found 697.3544.

##### 3,6-*O*-Dinicotinoyl-panaxatriol (**24**)

White amorphous powder, yield 66.5 % after chromatography with ethyl acetate-petroleum ether (25:75); ^1^H NMR (CDCl_3_) *δ* 9.18 (1H, s, H-2′), 9.14 (1H, s, H-2′′), 8.71 (2H, brs, H-6′, H-6′′), 8.23 (2H, m, H-4′, H-4′′), 7.34 (2H, m, H-5′, H-5′′), 3.54 (1H, d, *J* = 4.2 Hz, H-3), 1.22 (3H, s, H-26), 1.18 (3H, s, H-27), 1.15 (6H, s, H-28, H-30), 1.09 (6H, s, H-21, H-18), 1.06 (3H, s, H-19), 0.90 (3H, s, H-29). ^13^C NMR (CDCl_3_) *δ* 164.8 (CO, C-1′), 164.4 (CO, C-1′′), 153.3 (C, C-2′), 153.2 (C, C-2′′), 151.0 (CH, C-6′), 150.7 (CH, C-6′′),137.2 (CH, C-4′), 137.0 (CH, C-4′′), 126.4 (CH, C-3′), 126.4 (CH, C-3′′), 123.3 (CH, C-5′), 123.3 (CH, C-5′′), 81.4 (C-3), 76.5 (C, C-20), 73.1 (C, C-25), 71.9 (CH, C-12), 69.5 (C-6), 58.5 (C-5), 54.5 (CH, C-17), 51.0 (C, C-14), 49.2 (CH, C-9), 48.7 (CH, C-13), 42.6 (CH_2_, C-7), 40.8 (C, C-8), 39.4 (C, C-4), 38.1 (CH_2_, C-1), 38.1 (C, C-10), 36.3 (CH_2_, C-24), 35.6 (CH_2_, C-22), 32.9 (CH_3_, C-26), 31.0 (CH_2_, C-26), 30.8 (CH_3_, C-28), 30.3 (C-11), 27.1 (CH_3_, C-27), 25.0 (CH_2_, C-2), 23.3 (CH_2_, C-16), 19.3 (CH_3_, C-21), 17.2 (CH_3_, C-18), 17.1 (CH_3_, C-19), 17.0 (CH_3_, C-30), 16.8 (CH_3_, C-29), 16.2 (CH_2_, C-23). ESIMS(+): *m*/*z* 687 [M+H]^+^, HRESIMS: calcd for C_42_H_59_N_2_O_6_ [M+H]^+^ 687.4368, found 687.4332.

##### 3,6-*O*-Di(2′-methoxyl)acetoxyl-panaxatriol (**25**)

White amorphous powder, yield 74.9 % after chromatography with ethyl acetate-petroleum ether (10:90); ^1^H NMR (CDCl_3_) *δ* 5.39 (1H, dd, *J* = 9.9, 6.6 Hz, H-6). 4.52 (1H, d, *J* = 10.9, 5.1 Hz, H-3), 3.97 (4H, s, H-2′, H-2′′), 3.91 (1H,td, *J* = 10.3, 5.2 Hz, H-12), 3.38 (6H, s, H-3′, H-3′′), 1.20 (3H, s, H-27), 1.14 (3H, s, H-26), 1.11 (3H, s, H-21), 1.06 (3H, s, H-18), 0.96 (3H, s,H-28), 0.94 (3H, s, H-19), 0.85 (3H, s, H-29), 0.82 (3H, s, H-30). ^13^C NMR (CDCl_3_) *δ* 164.8 (CO, C-1′), 164.4 (CO, C-1′′), 153.3 (C, C-2′), 153.2 (C, C-2′′), 151.0 (CH, C-6′), 150.7 (CH, C-6′′),137.2 (CH, C-4′), 137.0 (CH, C-4′′), 126.4 (C, C-3′), 126.4 (C, C-3′′), 123.3 (CH, C-5′), 123.3 (CH, C-5′′), 81.4 (C-3), 76.5 (C, C-20), 73.1 (C, C-25), 71.9 (CH, C-12), 69.5 (C-6), 58.5 (C-5), 54.5 (CH, C-17), 51.0 (C, C-14), 49.2 (CH, C-9), 48.7 (CH, C-13), 42.6 (CH_2_, C-7), 40.8 (C, C-8), 39.4 (C, C-4), 38.1 (CH_2_, C-1), 38.1 (C, C-10), 36.3 (CH_2_, C-24), 35.6 (CH_2_, C-22), 32.9 (CH_3_, C-26), 31.0 (CH_2_, C-26), 30.8 (CH_3_, C-28), 30.3 (C-11), 27.1 (CH_3_, C-27), 25.0 (CH_2_, C-2), 23.3 (CH_2_, C-16), 19.3 (CH_3_, C-21), 17.2 (CH_3_, C-18), 17.1 (CH_3_, C-19), 17.0 (CH_3_, C-30), 16.8 (CH_3_, C-29), 16.2 (CH_2_, C-23). ESIMS(+): *m*/*z* 621 [M+H]^+^, HRESIMS: calcd for C_36_H_61_O_8_ [M+H]^+^ 621.4361, found 621.4313.

#### Synthesise of Compounds **26**–**28**

Compounds **1**, **4** and **14**, were treated respectively with the Jones reagent (10 equiv.), in acetone (5 mL) at room temperature for 4 h. The reaction mixture was filtered and diluted with chloroform (50 mL). Then, the mixture was washed and concentrated by the method as mentioned above. The crude product was processed by the silica gel column chromatography.

##### (20*R*)-20,25-Epoxy-3-*O*-acetoyl-dammaran-12-dione (**26**)

White amorphous powder, yield 63.8 % after chromatography with acetone-petroleum ether (10:90); ^1^H NMR (CDCl_3_) *δ* 4.47 (1H, dd, *J* = 11.3, 5.1 Hz, H-3*α*), 2.03 (3H, s, H-2′), 1.18 (6H, s), 1.16 (3H, s, H-27), 1.09 (3H, s, H-21),0.96 (3H, s, H-18), 0.87 (3H, H-29), 0.85 (3H, H-19), 0.72 (3H, H-30). ^13^C NMR (CDCl_3_) *δ* 212.3 (CH, C-12), 170.8 (CO, C-1′), 80.4 (CH, C-3), 74.7 (C, C-25), 70.6 (C, C-20), 56.1 (CH, C-13), 55.8 (CH, C-5), 55.6 (C, C-14), 54.3 (CH, C-9), 45.9 (CH, C-17), 40.3 (C, C-4), 39.8 (CH_2_, C-11), 38.2 (CH_2_, C-1), 38.2 (C, C-8), 37.8 (C, C-10), 37.5 (CH_2_, C-24), 34.1 (CH_2_, C-22), 33.6 (CH_3_, C-26), 33.4 (CH_2_, C-7), 32.2 (CH_2_, C-15), 27.9 (CH_3_, C-28), 27.3 (CH_3_, C-27), 25.8 (CH_3_, C-21), 24.0 (CH_2_, C-2), 23.5 (CH_2_, C-16), 21.3 (C, C-2′), 18.2 (CH_2_, C-6), 16.8 (CH_3_, C-30), 16.4 (CH_3_, C-29), 16.3 (CH_2_, C-23), 16.1 (CH_3_, C-19), 15.6 (CH_3_, C-18). ESIMS(+): *m*/*z* 501 [M+H]^+^, HRESIMS: calcd for C_32_H_53_O_4_ [M+H]^+^ 501.3938, found 501.3930.

##### (20*R*)-20,25-Epoxy-3-*O*-(2′-thenoyl)-dammaran-12-dione (**27**)

White amorphous powder, yield 49.1 % after chromatography with acetone-petroleum ether (10:90); ^1^H NMR (CDCl_3_) *δ* 7.72 (1H, dd, *J* = 3.7, 1.2 Hz, H-2′), 7.46 (1H, dd, *J* = 5.0, 1.2 Hz, H-4′), 7.03 (1H, dd, *J* = 5.0, 3.7 Hz, H-3′), 4.59 (1H, dd, *J* = 11.6, 4.9 Hz, H-3*α*), 3.00 (1H, d, *J* = 8.9 Hz, H-13*β*), 1.13 (3H, s, H-27), 1.12 (3H, s, H-26), 1.10 (3H, s, H-21), 1.03 (3H, s, H-18), 0.93 (3H, H-28), 0.93 (3H, H-19), 0.87 (3H, H-29), 0.67 (3H, H-30). ^13^C NMR (CDCl_3_) *δ* 212.3 (CH, C-12), 161.9 (CO, C-1′), 134.5 (C, C-2′), 133.1 (C, C-3′), 132.1 (CH, C-4′), 127.7 (CH, C-5′), 81.4 (CH, C-3), 74.7 (C, C-25), 70.7 (C, C-20), 56.2 (CH, C-13), 55.8 (CH, C-5), 55.7 (C, C-14), 54.3 (CH, C-9), 46.0 (CH, C-17), 40.4 (C, C-4), 39.8 (CH_2_, C-11), 38.3 (CH_2_, C-1), 37.6 (C, C-8), 37.0 (C, C-10), 34.2 (CH_2_, C-24), 33.6 (CH_3_, C-26), 33.5 (CH_2_, C-22), 33.4 (CH_2_, C-7), 32.2 (CH_2_, C-15), 28.1 (CH_3_, C-28), 27.4 (CH_3_, C-27), 25.8 (CH_3_, C-21), 24.1 (CH_2_, C-2), 24.0 (CH_2_, C-16), 18.3 (CH_2_, C-6), 16.9 (CH_3_, C-30) 16.6 (CH_3_, C-29), 16.4 (CH_2_, C-23), 16.1 (CH_3_, C-19), 15.6 (CH_3_, C-18). ESIMS: *m/z* 569 [M+H]^+^, HRESIMS: calcd for C_35_H_53_O_4_S [M+H]^+^ 569.3659, found 569.3662.

##### (20*R*)-20,25-Epoxy-3-*O*-succinyl-dammaran-12-dione (**28**)

White amorphous powder, yield 63.8 % after chromatography with acetone-petroleum ether (10:90); ^1^H NMR (CDCl_3_) *δ* 4.45 (1H, dd, *J* = 11.6, 4.8 Hz, H-3*α*), 1.19 (3H, s, H-26), 1.12 (6H, s, H-28, H-27), 1.03 (3H, s, H-21), 0.90 (3H, s, H-30), 0.81 (3H, s, H-29), 0.79 (3H, s, H-19), 0.66 (3H, s, H-18). ^13^C NMR (CDCl_3_) δ: 212.3 (CH, C-12), 177.2 (CH, C-4′), 171.8 (CO, C-1′), 81.0 (CH, C-3), 74.7 (C, C-25), 70.7 (C, C-20), 56.2 (CH, C-13), 55.8 (CH, C-5), 55.8 (C, C-14), 54.3 (CH, C-9), 45.9 (CH, C-17), 40.4 (C, C-4), 39.8 (CH_2_, C-11), 38.2 (CH_2_, C-1), 37.9 (C, C-8), 37.6 (C, C-10), 37.0 (CH_2_, C-24), 34.2 (CH_2_, C-22), 33.6 (CH_3_, C-26), 33.5 (CH_2_, C-7), 32.2 (CH_2_, C-15), 29.3 (C, C-3′), 28.9 (C, C-2′), 27.9 (CH_3_, C-28), 27.4 (CH_3_, C-27), 25.8 (CH_3_, C-21), 24.1 (CH_2_, C-2), 23.5 (CH_2_, C-16), 18.3 (CH_2_, C-6), 16.8 (CH_3_, C-30), 16.5 (CH_3_, C-29), 16.4 (CH_2_, C-23), 16.1 (CH_3_, C-19), 15.6 (CH_3_, C-18). ESIMS: *m/z* 559 [M+H]^+^, HRESIMS: calcd for C_34_H_55_O_6_ [M+H]^+^ 559.3969, found 559.4000.

### In Vitro Anti-HBV Assay

Based on our previous description [9], inhibitory activity on HBV (HBsAg, HBeAg and HBV DNA) was evaluated. The anti-HBV activities and cytotoxicity of compounds were observed on the HepG 2.2.15 cells. Cytotoxicity was assayed with a modified 3-(4,5-dimethylthiazole-2-yl)-2,5-diphenyltetrazolium bromide (MTT) method (Gibco Invitrogen, Carlsbad, CA, USA). The anti-HBV antigen secretion activities were determined by the enzyme linked immunosorbent assay (ELISA; Autobio Diagnostics Co., Ltd, China). A real-time PCR assay was applied to detect the inhibitory activity on HBV DNA replication.

## Electronic supplementary material

Below is the link to the electronic supplementary material.

Supplementary material 1 (DOCX 2468 kb)
