# Supplementary material for: Panaxadiol and Panaxatriol Derivatives as Anti-Hepatitis B Virus Inhibitors
Source: Nat Prod Bioprospect. 2014 May 13;4(3):163–74. doi: 10.1007/s13659-014-0018-2 (PMC4050313; doi:10.1007/s13659-014-0018-2)
Supplement: Supplementary file 1 — Supplementary material 1 (DOCX 2468 kb) [file 13659_2014_18_MOESM1_ESM.docx]

**Panaxadiol and panaxatriol derivatives as anti-hepatitis B virus inhibitors**

Hao Chen ^a, b^, Li-Jun Wang ^a^_,_ Yun-Bao Ma ^a^, Xiao-Yan Huang ^a^, Chang-An Geng ^a^, Xue-Mei Zhang ^a^, and Ji-Jun Chen ^a*^

*^a^State Key Laboratory of Phytochemistry and Plant Resources in West China, Kunming Institute of Botany, Chinese Academy of Sciences, Kunming 650201, PR China*

*^b^University of Chinese Academy of Sciences, Beijing 100049, PR China*

**Supporting Information List**

**Characterization Data of New Compounds**

Page 2: ^1^H NMR spectrum of compound **4**

Page 3: ^13^C NMR (DEPT) spectrum of compound **4**

Page 4: ^1^H NMR spectrum of compound **6**

Page 5: ^13^C NMR (DEPT) spectrum of compound **6**

Page 6: ^1^H NMR spectrum of compound **14**

Page 7: ^13^C NMR (DEPT) spectrum of compound **14**

Page 8: ^1^H NMR spectrum of compound **15**

Page 9: ^13^C NMR (DEPT) spectrum of compound **15**

Page 10: ^1^H NMR spectrum of compound **16**

Page 11: ^13^C NMR (DEPT) spectrum of compound **16**


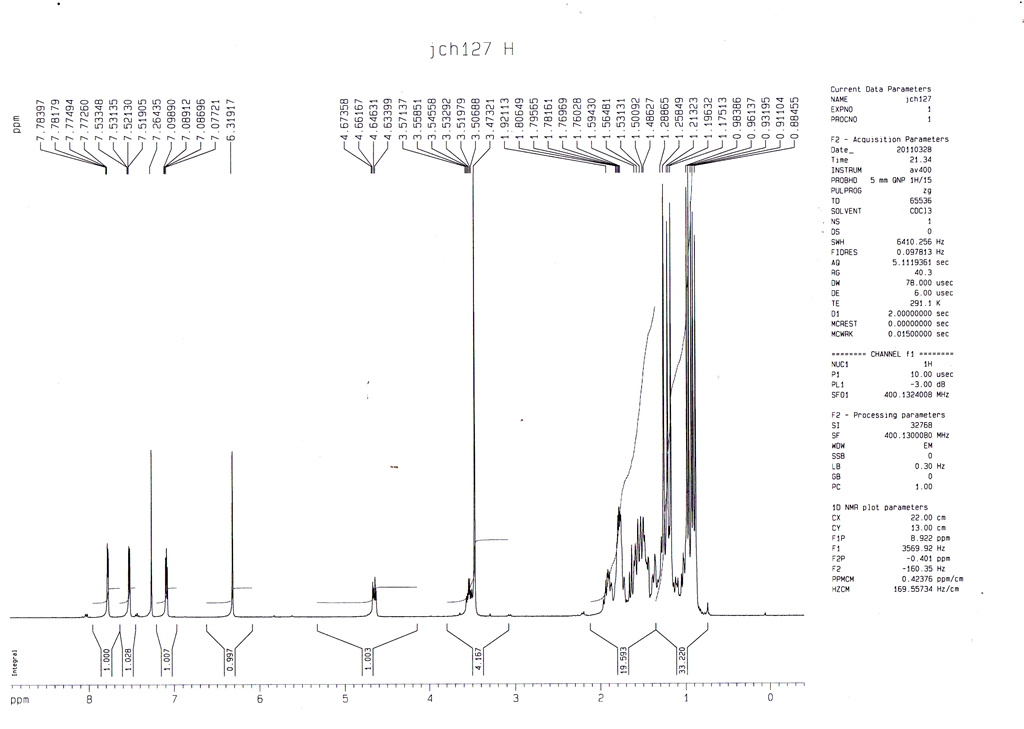

**Spectra 1** ^1^H NMR spectrum of compound **4** (400MHz, CD_3_OD)


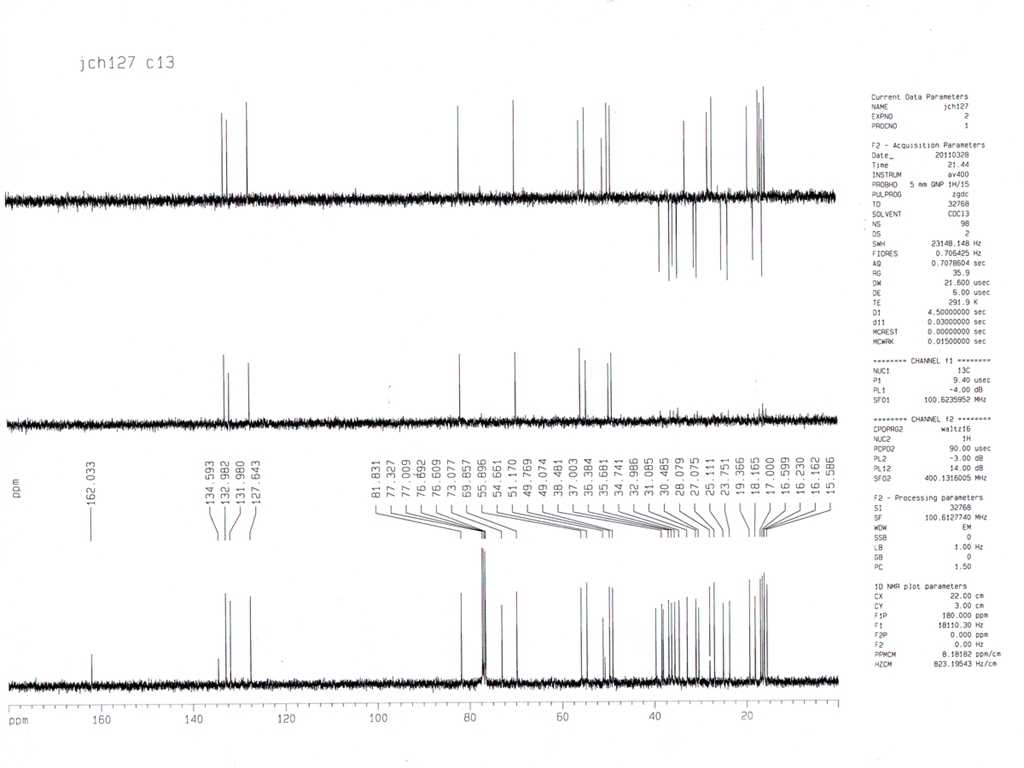

**Spectra 1** ^13^C NMR spectrum of compound **4** (100MHz, CD_3_OD)


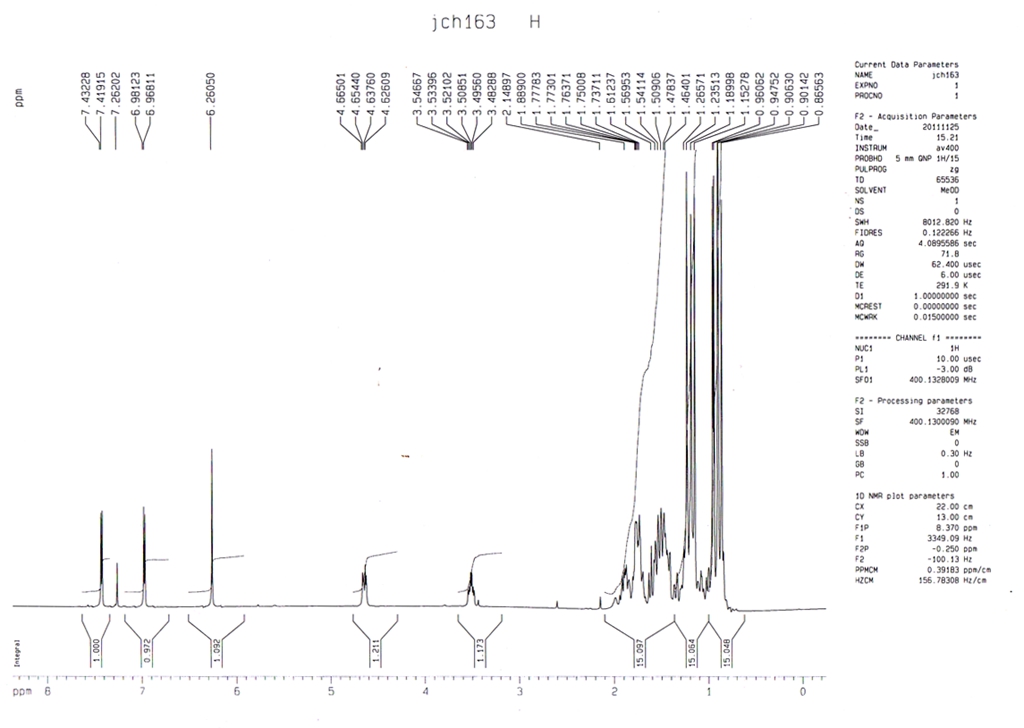

**Spectra 2** ^1^H NMR spectrum of compound **6** (400MHz, CD_3_OD)


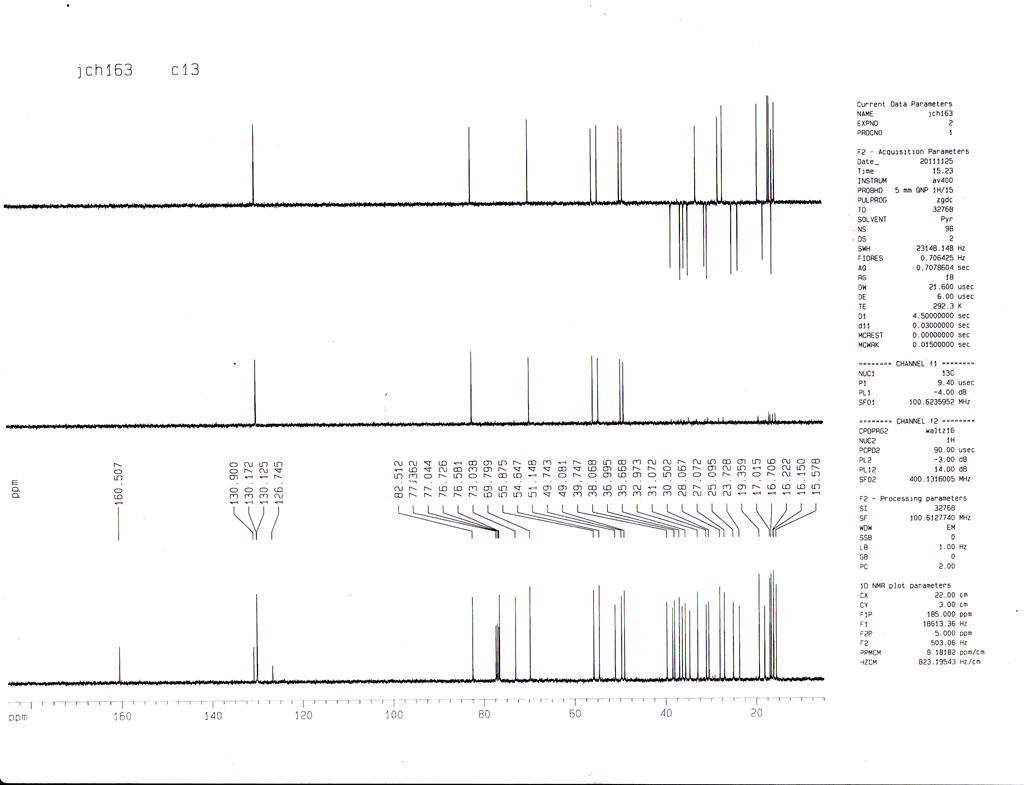

**Spectra 2** ^13^C NMR spectrum of compound **6** (100MHz, CD_3_OD)


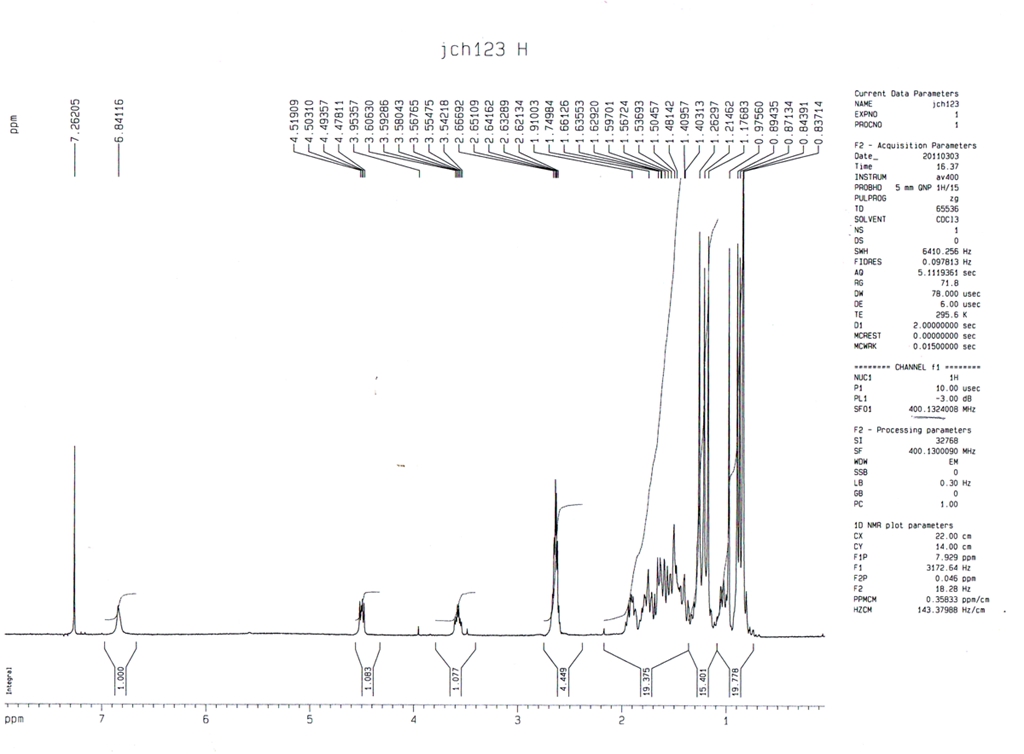

**Spectra 3** ^1^H NMR spectrum of compound **14** (400MHz, CD_3_OD)


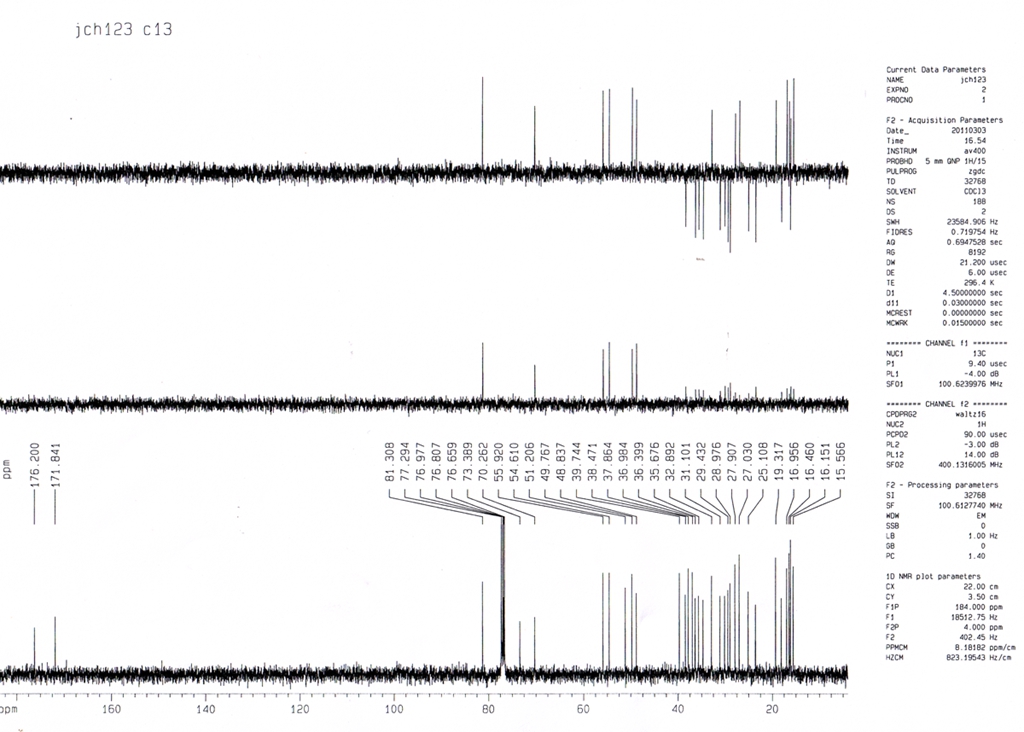

**Spectra 3** ^13^C NMR spectrum of compound 1**4** (100MHz, CD_3_OD)


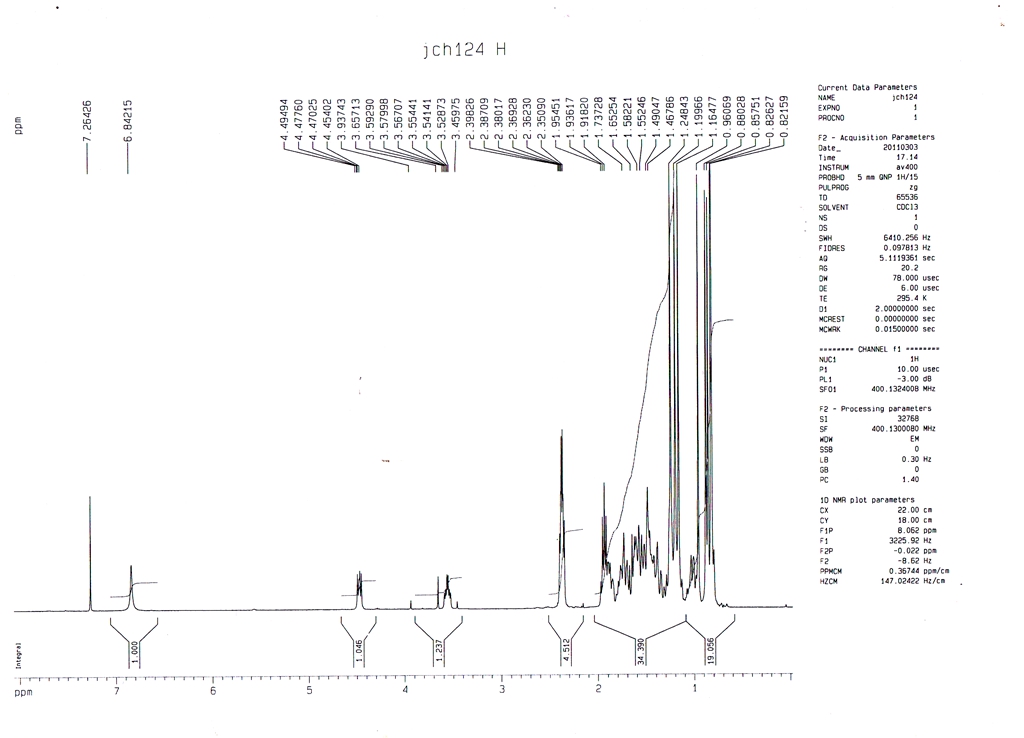

**Spectra 4** ^1^H NMR spectrum of compound **15** (400MHz, CD_3_OD)


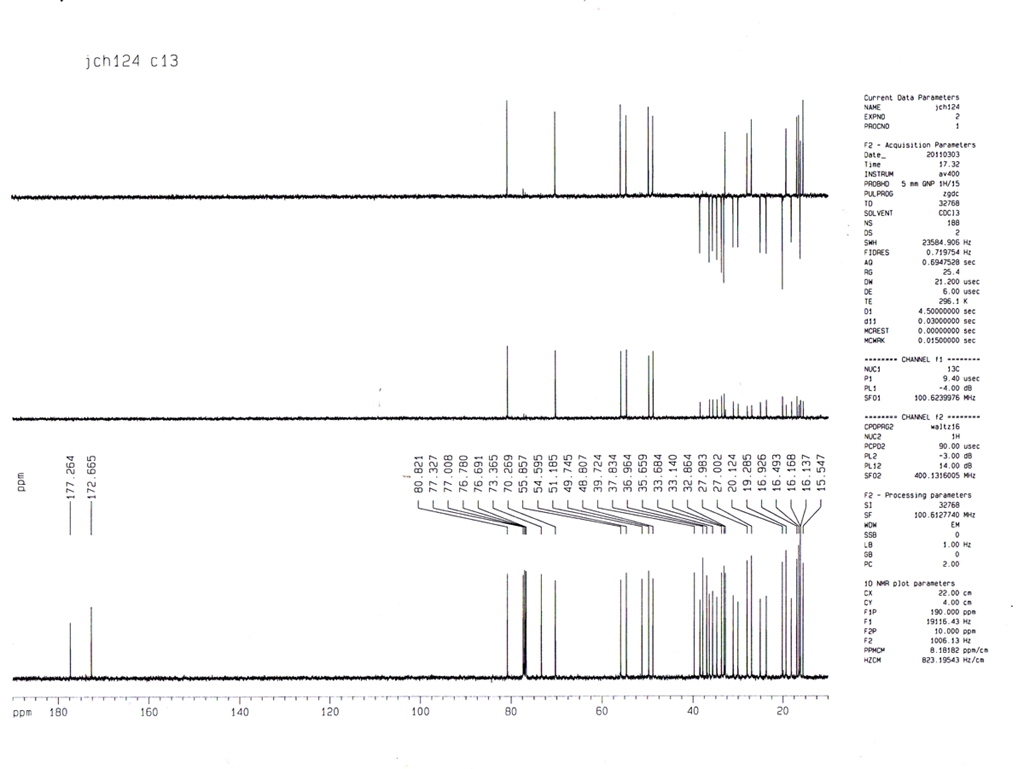

**Spectra 4** ^13^C NMR spectrum of compound **15** (100MHz, CD_3_OD)


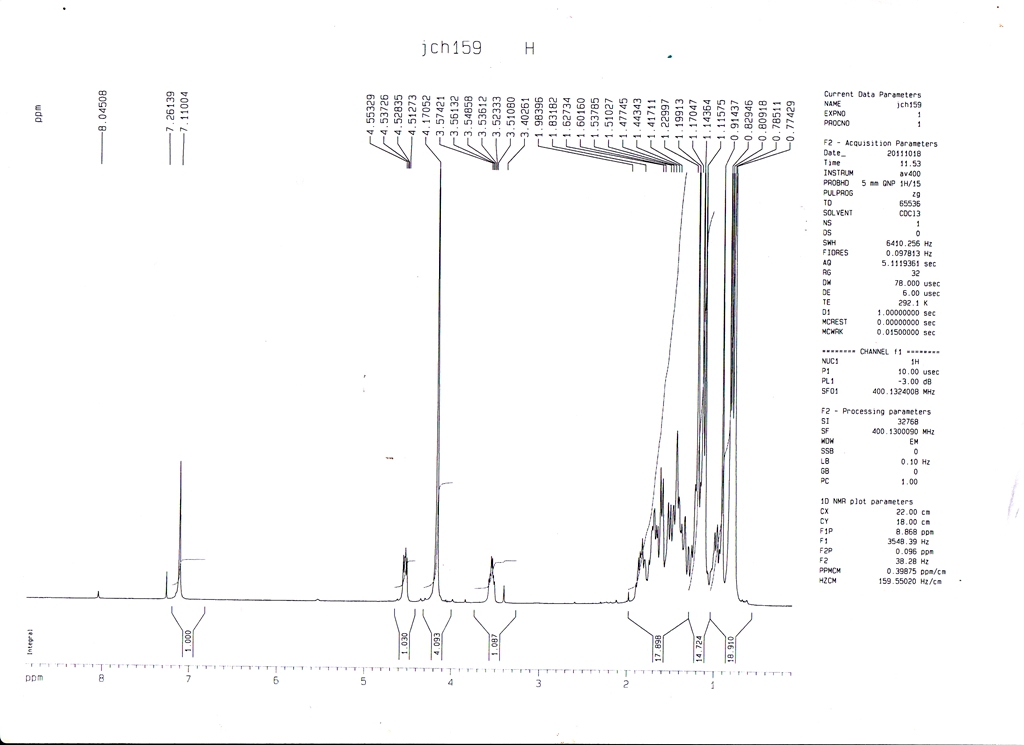

**Spectra 5** ^1^H NMR spectrum of compound **16** (400MHz, CD_3_OD)


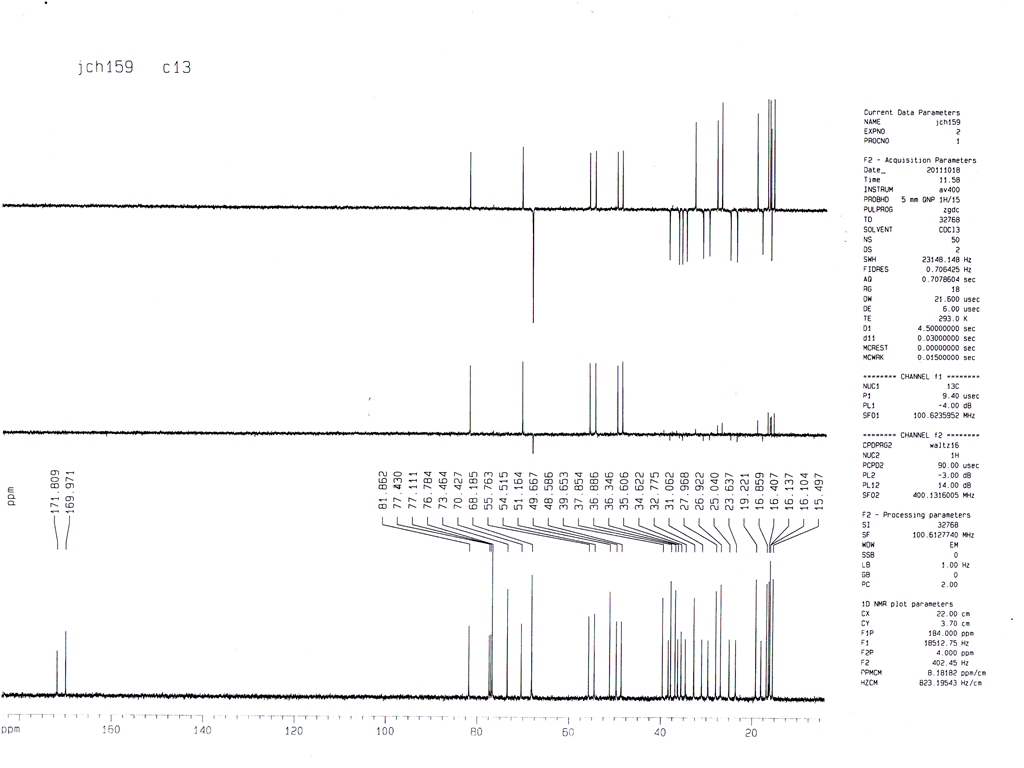

**Spectra 5** ^13^C NMR spectrum of compound **16** (100MHz, CD_3_OD)
